# A deep learning framework for lysine 2-hydroxyisobutyrylation site prediction using evolutionary feature representation

**DOI:** 10.1038/s41598-025-15883-z

**Published:** 2025-11-06

**Authors:** Heba M. Elreify, Fathi E. Abd El-Samie, Moawad I. Dessouky, Hanaa Torkey, Said E. El-Khamy, Wafaa A. Shalaby

**Affiliations:** 1 Department of Electronics and Electrical Communication Engineering, Faculty of Electronic Engineering, Menoufia University, Menouf, 32952 Egypt; 2 Department of Information Technology, College of Computer and Information Sciences, Princess Nourah Bint Abdulrahman University, P.O. Box 84428, Riyadh, 11671 Saudi Arabia; 3 Department of Computer Science and Engineering, Faculty of Electronic Engineering, Menoufia University, Menouf, 32952 Egypt; 4 Department of Computer Science, College of Computer Engineering and Sciences, Prince Sattam Bin Abdulaziz University, Al-Kharj, 16273 Saudi Arabia; 5 Department of Electrical Engineering, Faculty of Engineering, Alexandria University, Alexandria, Egypt

**Keywords:** Post-translational modifications, Deep learning, Blosum62, Lysine 2-hydroxyisobutyrylation, Cross-species, Post-translational modifications, Computational biology and bioinformatics

## Abstract

Lysine 2-hydroxyisobutyrylation (Khib) has emerged as a crucial Post-Translational Modification (PTM) with significant roles in diverse biological processes ranging from gene expression to metabolic regulation. Despite its importance, computational approaches for accurately predicting Khib sites remain limited. This study introduces BLOS-Khib, a deep-learning framework that utilizes evolutionary information encoded in the BLOSUM62 matrix within a Convolutional Neural Network (CNN) architecture for cross-species Khib site prediction. Through systematic optimization, we found that a 43-amino acid peptide length captures the optimal sequence context for prediction across six taxonomically diverse organisms. Comprehensive comparative analyses demonstrated BLOS-Khib competitive performance compared to existing methods, achieving notable Area Under the ROC Curve (AUC) values on independent test sets: human (0.913), wheat (0.892), *T. gondii* (0.893), rice (0.887), *Candida albicans* (0.885), and *Botrytis cinerea* (0.903). Our framework showed improved performance compared to state-of-the-art approaches, including traditional machine learning classifiers and alternative deep learning architectures. Sequence signature analysis revealed both conserved lysine-rich regions preceding modification sites and species-specific amino acid preferences at positions immediately flanking the target residue. Notably, our cross-species applicability experiments identified high transferability between evolutionarily distant organisms, ensuring the potential convergent evolution of Khib determinants. BLOS-Khib demonstrates competitive performance for PTM prediction, while providing evolutionary insights into the sequence determinants governing this emerging regulatory mechanism across diverse species.

**Supplementary Information:**

The online version contains supplementary material available at 10.1038/s41598-025-15883-z.

## Introduction

The PTMs are critical biochemical events that occur after protein synthesis, significantly enhancing the functional diversity of the proteome beyond the primary amino acid sequence^1^. These covalent alterations, such as phosphorylation, acetylation, methylation, ubiquitination, and glycosylation, modulate protein activity, stability, localization, and interactions, thereby regulating a wide array of cellular processes^2,3^. Dysregulation of PTMs is implicated in numerous diseases, including cancer, neurodegeneration, and metabolic disorders, positioning PTM sites as valuable targets for therapeutic intervention^4,5^.

Among PTMs, lysine modifications have received particular attention due to their regulatory significance. In addition to well-studied forms like acetylation and methylation, newer modifications such as crotonylation, malonylation, and Khib have been identified^6^. Khib, first reported by Tan et al. in 2014 through mass spectrometry-based proteomics, was initially observed on histones and linked to active gene transcription^7^. The modification introduces a 2-hydroxyisobutyryl group (+ 86.037 Da) to lysine ε-amino group, neutralizing its charge and potentially influencing protein-DNA and protein-protein interactions^8^.

Khib has since been identified across a broad spectrum of organisms, from bacteria to plants and mammals, underscoring its evolutionary conservation and biological relevance^9^. Despite the utility of mass spectrometry for experimentally identifying PTM sites, it faces challenges such as technical complexity, low stoichiometry of modifications, and incomplete coverage due to dynamic PTM behaviour^10^. These limitations highlight the need for complementary computational methods that can efficiently predict PTM sites across whole proteomes, especially in species lacking experimental data^11^.

Deep learning has revolutionized computational PTM site prediction by enabling the capture of complex sequence dependencies. Architectures such as CNNs, Long Short-Term Memory (LSTM) networks, and transformer-based models have been widely adopted for their ability to learn biologically meaningful patterns from raw protein sequences^12^. Tools like MusiteDeep^13^, DeepPhos^14^, and DeepUbi^15^ have demonstrated the effectiveness of CNNs for phosphorylation and ubiquitination site prediction across a range of species. Hybrid and transfer learning approaches like EMBER^16^, MDC-Kace^17^, and DeepTL-Ubi^18^ have further advanced the field by integrating multiple feature types and enabling cross-species predictions.

Recent advances have demonstrated the power of sophisticated architectures for various PTM predictions. Notable examples include transformer-based models that can capture long-range dependencies in protein sequences and ensemble approaches that combine multiple architectural paradigms^19^. The development of PTM-Mamba, a state-of-the-art protein language model specifically trained on PTM-labelled data, represents a significant advancement in the field, demonstrating how specialized pre-training can enhance PTM prediction capabilities^20^. Additionally, comprehensive benchmarking efforts such as UniPTM have established frameworks for evaluating multiple PTM site prediction methods on full-length protein sequences, providing standardized evaluation protocols for the community^21^.

Efforts specific to lysine PTMs have also gained momentum. For instance, SEMal and other methylation predictors incorporate evolutionary and structural data to boost accuracy^22,23^. In the Khib domain, several tools have been developed, beginning with KhibPred, the first Khib predictor utilized an ensemble Support Vector Machine (SVM) approach, combining the composition of k-spaced amino acid pairs, binary encoding, and amino acid factors, to achieve an AUC of 0.7937 on a dataset of 4,659 Khib sites from 1,496 proteins^24^. iLys-Khib refined this approach using fuzzy SVMs and feature selection, achieving 70.12% accuracy but revealing limitations in sensitivity-specificity balance and general predictive power^25^.

The transition to deep learning approaches marked a significant advancement in Khib prediction capabilities. DeepKhib applied CNNs with one-hot encoding and demonstrated high performance (AUC 0.82–0.87) across five species, though its general model underperformed compared to species-specific ones^26^. ResNetKhib introduced residual networks with word embeddings and cell type-specific predictions, improving cross-context prediction (AUC 0.807–0.901), but evolutionary information remained underutilized^27^.

Despite these advances, several fundamental limitations persist across existing Khib prediction tools. Most existing tools are trained on relatively constrained datasets, often focused on human proteins, with limited systematic validation across taxonomically diverse species. Furthermore, given that Khib is a relatively recent discovery, the computational prediction landscape remains underexplored, with only a few predictors available. There is still substantial room for improvement in predictive accuracy and generalizability. Moreover, the ongoing discovery of novel Khib sites in newly studied species underscores the need for more adaptable and biologically grounded predictive frameworks.

In this study, we propose BLOS-Khib, a deep-learning framework that leverages evolutionary information encoded in BLOcks SUbstitution Matrix (BLOSUM62) through a one-dimensional CNN (1DCNN) architecture. This approach aims to enhance Khib site prediction by capturing biologically meaningful patterns of residue substitutions.

The main contributions of this work are as follows: (i) development of BLOS-Khib, a 1DCNN-based architecture optimized using BLOSUM62 encoding; (ii) comparison of six feature representation techniques across six taxonomically diverse organisms; (iii) optimization of input window size, identifying a 43-residue context as being optimal for Khib site prediction; (iv) benchmarking against alternative deep learning architectures and classical machine learning classifiers; (v) evaluation of cross-species model generalizability; and (vi) sequence motif analysis to reveal conserved and species-specific features surrounding Khib sites. The remainder of this paper is organized as follows. The next section presents our methods. After that, the results are presented. Finally, the last section summarizes our findings, limitations, and future research directions.

## Methods

**Fig. 1:**
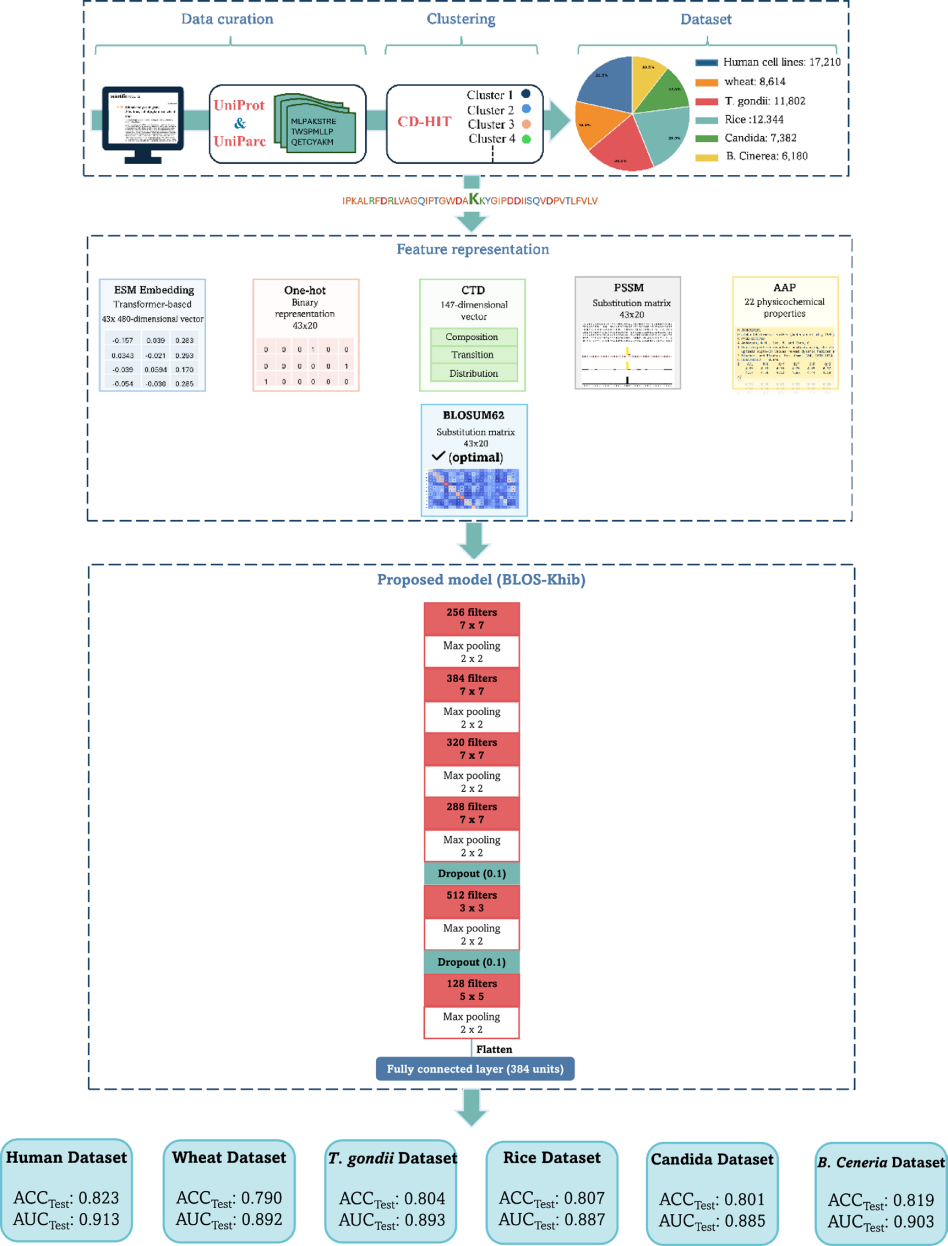
Comprehensive workflow for Khib site prediction: data curation, feature representation strategies, and architecture of the proposed BLOS-Khib model compared with alternative deep learning approaches.

Figure 1 illustrates that the workflow encompasses three primary components: (i) data curation from six taxonomically diverse organisms, followed by clustering to reduce sequence redundancy; (ii) implementation and evaluation of multiple feature representation strategies with particular emphasis on BLOSUM62 matrix encoding; and (iii) development of a specialized 1DCNN architecture (BLOS-Khib).

### Dataset

To effectively predict Khib sites, we compiled and curated diverse datasets from multiple organisms with experimentally verified Khib sites. These organisms include human cell lines (HeLa, lung, and pancreatic cancer cells)^28–30^, *Triticum aestivum* (common wheat and wheat root)^31,32^, *Toxoplasma gondii* (ME49 and RH strains)^33^, *Oryza sativa* (rice seeds and leaves)^34,35^, Candida albicans^36^, and Botrytis cinerea^37^. For simplicity, we will refer to these organisms as wheat (*Triticum aestivum*), *T. gondii* (*Toxoplasma gondii*), rice (*Oryza sativa*), Candida (*Candida albicans*), and *B. cinerea* (*Botrytis cinerea*) throughout this study.

For each organism, we developed a Python script to directly extract peptide sequences of the required length, centred on Khib-modified lysine residues. This script utilized protein accession numbers and specific lysine position information from published studies to query the UniProt database and extract target lysines with their surrounding amino acids.

To determine the optimal peptide length, we systematically evaluated window sizes ranging from 35 to 47 amino acids using the human dataset as a representative model. The 43-residue window (21 residues flanking the central lysine on each side) yielded the highest AUC value during 10-fold cross-validation and was therefore selected for all subsequent analyses. For lysine residues located near protein termini with insufficient flanking residues, the symbol “X” was used as padding to maintain uniform sequence length.

Positive samples were generated by extracting 43 amino acid peptide fragments centred on experimentally validated Khib-modified lysine residues. Each positive sample represents a confirmed Khib modification site with its surrounding sequence context. Negative samples were generated using the same proteins containing positive samples, extracting peptides centred on lysine residues where no Khib modification was experimentally detected. This approach yielded substantially larger pools of potential negative samples compared to positive ones, creating an inherent class imbalance that required careful handling.

To address sequence redundancy and class imbalance, we employed the Cluster Database at High Identity with Tolerance (CD-HIT) tool^38^ with a 40% sequence similarity threshold. This clustering approach groups sequences sharing greater than 40% similarity, retaining one representative sequence from each cluster to eliminate redundant training examples. The 40% similarity threshold was selected based on established protocols in PTM prediction research^23,27,39^, which have shown this threshold to optimally balance dataset diversity while ensuring sufficient training examples for robust model development.

The clustering process was applied to both positive and negative samples, separately. Following clustering, negative clusters were randomly selected to match the number of positive clusters, creating balanced datasets with 1:1 positive-to-negative ratios. This balancing approach follows established methodologies in PTM prediction research^40–42^ and specifically in Khib prediction studies^26^, effectively mitigating class imbalance bias while preserving biological relevance and sequence diversity.

Table 1 presents the final dataset composition after clustering and balancing. The clustering process substantially reduced dataset redundancy across all organisms. For example, the human dataset was reduced from 25,676 to 8,605 positive clusters. Each species-specific dataset was partitioned into training (90%) and independent test (10%) sets. Importantly, to ensure rigorous model validation and avoid overestimation of predictive performance, the clustering was performed before data splitting. This ensures no highly similar sequences in both training and test sets.

The training set underwent 10-fold cross-validation for model optimization and hyperparameter tuning, providing stable performance estimates and ensuring robust model development. The independent test set was reserved exclusively for the final performance assessment, serving as an unbiased evaluation of model generalization capacity. A general multi-organism dataset comprising 63,532 samples from all six organisms was also created to develop cross-species prediction models and evaluate transferability across taxonomic boundaries.

**Table 1:** Distribution of Khib sites across multiple organisms.

Dataset		Number of collected positive samples	Number of proteins	Number of positive clusters	Total samples
Human	Hela cells	6,544	1,725	8,605	17,210
	Lung cells	8,765	2,484		
	Pancreatic cancer cells	10,367	2,325		
Wheat	Common wheat	3,348	1,047	4,307	8,614
	Wheat root	6,328	2,186		
*Toxoplasma gondii*	ME49	8,092	1,720	5,901	11,802
	RH	9,502	1,950		
Rice	Rice seeds	9,916	2,512	6,172	12,344
	Rice leaves	4,163	1,596		
*Candida albicans*		6,659	1,438	3,691	7,382
*Botrytis cinerea*		5,398	1,181	3,090	6,180
General (multi-organism)					63,532

### Feature representation methods

Accurate prediction of PTM sites relies heavily on the effective numerical representation of protein sequence information. In this study, we implemented multiple complementary feature encoding methods to capture different aspects of protein sequences relevant to Khib modification. These methods include: (1) embedding-based method that leverages deep learning models to capture complex patterns; (2) context-based method that represents the local sequence environment; (3) sequence-based method that encodes compositional and distributional patterns of physicochemical properties; (4) physicochemical property-based method that directly incorporates numerical indices representing diverse amino acid properties such as hydrophobicity, volume, and charge; and (5) evolutionary methods that integrate conservation information through substitution matrices. Each representation method provides a unique perspective on the protein sequence characteristics that may influence Khib site occurrence. The following sections detail all feature representation methods employed in our study.

#### Embedding-based method

Evolutionary Scale Modeling (ESM) is an advanced transformer-based protein language model that effectively captures the evolutionary, structural, and functional characteristics of protein sequences^43^. It employs multi-head self-attention mechanisms that model both local residue interactions and long-range dependencies, enabling the extraction of context-sensitive embeddings that reflect evolutionary conservation and residue environments^44^.

The ESM-2 architecture enhances its predecessor, ESM-1b^45^, through refined attention mechanisms and training methodologies that improve biological representation learning. The model undergoes pretraining on UniRef50^46^, which clusters protein sequences at 50% identity to ensure diversity while reducing redundancy, thereby enabling the model to learn generalizable principles of protein evolution.

To generate embeddings, ESM tokenizes each amino acid sequence and passes it through multiple transformer layers, where self-attention captures residue relationships across the sequence^47^. During the forward pass, attention weights highlight conserved regions, functional motifs, and structurally important residues. The final hidden states serve as per-residue embedding representations.

ESM produces two types of embeddings: per-residue embeddings, which provide context-aware vectors for individual amino acids (ranging from 320 to 5120 dimensions depending on the variant); and fixed embeddings, which offer global sequence-level representations suitable for classification or comparative analyses. In our implementation, protein sequences were processed through the pre-trained ESM-2 model to obtain per-residue embeddings, with each residue encoded as a high-dimensional vector encompassing physicochemical properties, secondary structures, and functional motifs.

The ESM model family includes variants of different sizes and capacities, denoted as *esm2_t*{*layers*} _{*parameters*}_*UR50D*, where {*layers*} refers to the number of transformer layers and {*parameters*} represents the total model size. Larger variants demand more computational resources without necessarily offering better performance for specific tasks. To determine the optimal variant for Khib site prediction, we evaluated multiple ESM versions using the human dataset, selected for its large sample size and diverse cellular context, providing a robust benchmark for model performance.

To identify the most suitable ESM variant for Khib site prediction, we conducted extensive evaluations using the human dataset, prioritizing models up to 1280 embedding dimensions due to hardware limitations. As detailed in Table 2, esm2_t12_35M_UR50D achieved the best AUC scores for both 10-fold cross-validation (0.793) and independent testing (0.784), outperforming larger variants that offered no performance gains despite higher complexity. Consequently, we selected the ESM-2-35M model for its optimal trade-off between accuracy and efficiency. Each protein sequence was encoded into a 43 × 480 matrix of per-residue embeddings, effectively capturing evolutionary, structural, and functional context.

**Table 2:** Performance comparison of ESM2 model variants with embedding dimensions and computational requirements on the human dataset.

Model	Embedding dimension	Embedding Time (sec)	AUC_10 − CV_	AUC_Test_
esm2_t6_8m_UR50D	320	559	0.773	0.777
esm2_t12_35M_UR50D	480	842	0.793	0.784
esm2_t30_150m_UR50D	640	7,378	0.721	0.784
esm2_t33_650m_UR50D	1280	28,824	0.753	0.755
esm2_t36_3b_UR50D	2560	-	-	-
esm2_t48_15b_UR50D	5120	-	-	-

#### Context-based method

**One-hot encoding** was implemented as a baseline sequence-based representation method. In this method, each amino acid was represented as a 20-dimensional binary vector where only one element had a value of 1, corresponding to the specific amino acid. In contrast, all other elements are set to 0. For a sequence window of length 43, the one-hot encoded feature vector had a dimensionality of 43 × 20 = 860. This representation preserves the primary sequence information without incorporating any prior biological knowledge, serving as a fundamental baseline for comparative analysis.

#### Sequence-based method

**Composition**,** Transition**,** and Distribution (CTD)** descriptor was implemented to characterize the global and local distribution patterns of physicochemical properties within the sequence segments^48^. This method first categorized amino acids into three groups (1, 2, and 3) based on seven physicochemical properties: hydrophobicity, polarity, charge, polarizability, surface tension, secondary structure, and solvent accessibility. The Composition component gives the percentage frequency of each group within the sequence. The Transition component gives the frequency of transitions between different groups (e.g., from group 1 to group 2). The Distribution component represents the distribution patterns of each property group along the sequence by recording the positions of the first, 25%, 50%, 75%, and 100% occurrences of each group. This comprehensive encoding scheme resulted in a 147-dimensional feature vector (7 properties × (3 compositions + 3 transitions + 15 distributions)) that effectively captured the global sequence attributes and spatial arrangements of physicochemical properties relevant to lysine 2-hydroxyisobutyrylation.

#### Physicochemical property-based method

**Amino Acid Properties (AAP)** were derived from the AAindex database^49^, which contains over 566 numerical indices characterizing various physicochemical, biochemical, and structural properties of amino acids, as a foundation for our feature representation method. From this extensive repository, we carefully selected 22 indices based on their established relevance to protein functionality, structural characteristics, and potential involvement in post-translational modification mechanisms. The complete list of selected indices is provided in Table **S1**.

The selected properties encompass a diverse range of amino acid characteristics, including hydrophobicity indices that measure residue interactions with aqueous environments critical for protein folding and binding interactions; spatial parameters such as residue volume and bulkiness that quantify steric constraints; electronic properties, including polarizability and net charge that govern electrostatic interactions; and conformational flexibility metrics that reflect structural adaptability.

We also incorporated properties related to protein structure formation, including isoelectric point, secondary structure propensities such as alpha-helix frequency and coil conformation parameters, as well as solvent interaction measures, including solvation-free energy and hydration potential. This comprehensive set of properties provides a multidimensional characterization of the physicochemical environment surrounding potential 2-hydroxyisobutyrylation sites.

For feature encoding, we mapped each amino acid in our 43-residue peptide window to its corresponding values across all 22 selected indices. This process generated a feature vector with dimensions 43 × 22 = 946, where each position in the sequence was represented by 22 distinct physicochemical property values. This encoding strategy preserved both the positional context and the residue-specific properties, creating a rich feature representation that captures the complex biochemical landscape influencing lysine 2-hydroxyisobutyrylation.

#### Evolutionary methods

Evolutionary-based feature representation methods capture the conservation patterns and substitution preferences that have emerged through millions of years of evolution. These methods leverage evolutionary information to encode amino acid sequences in a biologically meaningful manner, providing insights into functional constraints and substitution tolerances at specific sequence positions.

**Position-Specific Scoring Matrix (PSSM)** profiles were generated to capture evolutionary conservation patterns within protein sequences^50^. For each protein sequence in our dataset, Position-Specific Iterative Basic Local Alignment Search Tool (PSI-BLAST)^51^ was performed against the Non-Redundant (NR) protein database with three iterations and an E-value threshold of 0.001. The resulting PSSM represented the log-likelihood of each amino acid occurring at each position in the sequence based on evolutionary conservation patterns.

The PSSM values quantify the degree of conservation for each amino acid at each position by measuring how frequently specific residues appear at corresponding positions in evolutionarily-related sequences. Positive scores indicate that a particular amino acid substitution occurs more frequently than expected by random chance, revealing evolutionary conservation and potential functional importance. Conversely, negative scores indicate substitutions that occur less frequently than expected, often representing evolutionarily-disfavored changes. A score of zero indicates that the substitution occurs at the background frequency expected by chance.

For our study utilizing a 43-amino acid peptide window, this resulted in a PSSM feature vector with dimensionality of 43 × 20 = 860 elements for each peptide sample. Each position in the 43-residue window was represented by 20 values corresponding to the substitution probabilities for all standard amino acids, thereby capturing the position-specific evolutionary constraints acting on the sequence surrounding potential Khib sites.

**BLOSUM** family represents a series of amino acid substitution matrices that quantify the likelihood of amino acid substitutions based on observed frequencies in evolutionarily related protein sequences^52^. BLOSUM62, the specific variant employed in this study, is derived from protein sequences sharing no more than 62% sequence identity, making it particularly suitable for detecting distant evolutionary relationships, while avoiding bias from highly-similar sequences.

Unlike position-specific methods such as PSSM, BLOSUM62 provides a general substitution probability framework between any two amino acids based on global evolutionary patterns observed across diverse protein families. This approach captures fundamental biochemical and evolutionary constraints that govern amino acid substitutions across all protein contexts. The BLOSUM62 matrix generation involves several key steps:

- Database construction: The matrix is derived from the BLOCKS database^53^, which contains multiple sequence alignments of conserved regions (blocks) from protein families. These blocks represent functionally important domains that are evolutionarily conserved across related proteins.
- Sequence clustering: Protein sequences within the BLOCKS database are clustered to eliminate redundancy, ensuring that sequences sharing more than 62% identity are grouped and represented by a single sequence.
- Substitution counting: Within each aligned block, all possible amino acid pairs at corresponding positions are counted to determine substitution frequencies. This process quantifies how often each amino acid is observed to substitute for every other amino acid in evolutionarily-related sequences.
- Log-Odds calculation: The observed substitution frequencies are compared to expected frequencies based on the background amino acid composition. The resulting log-odds scores represent the relative likelihood of each substitution occurring compared to random chance.
- Matrix normalization: The final matrix values are scaled and rounded to provide integer scores that facilitate computational efficiency, while preserving the underlying substitution relationships.

**Fig. 2:**
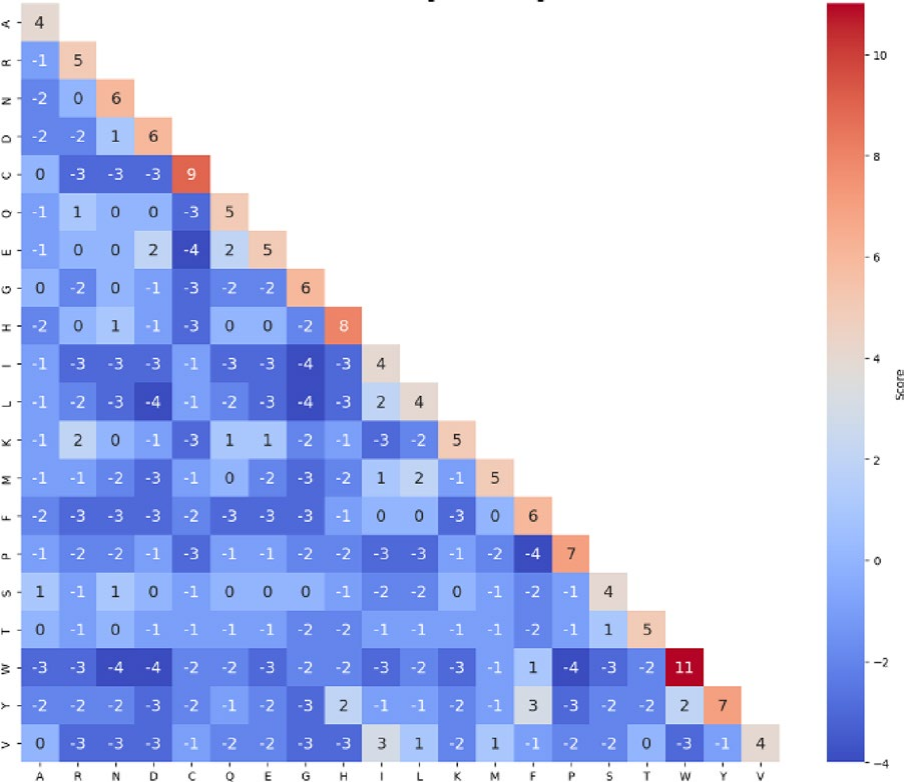
Visualization of the BLOSUM62 substitution matrix: highlighting amino acid conservation and substitution preferences in protein evolution.

As illustrated in Fig. 2, the BLOSUM62 matrix contains log-odds scores that quantify the likelihood of amino acid substitutions. Positive scores (displayed in red) indicate favourable substitutions that occur more frequently than expected by chance, reflecting evolutionary tolerance or preference for specific amino acid exchanges. Negative scores (displayed in blue) denote unfavourable substitutions that are evolutionarily rare, often due to functional or structural constraints. The diagonal elements of the matrix contain the highest positive values (ranging from 4 to 11), reflecting the strong evolutionary preference for amino acid conservation. For example, tryptophan (W) exhibits the highest conservation score of 11, indicating that tryptophan residues are highly conserved and rarely substituted during evolution, likely due to their unique structural properties and functional importance.

The BLOSUM62 encoding process transforms amino acid sequences into numerical feature vectors by utilizing evolutionary substitution patterns. For each amino acid position within the 43-residue peptide window, the corresponding row from the BLOSUM62 substitution matrix is retrieved, containing 20 values representing substitution scores between that specific amino acid and all standard amino acids. These substitution scores are concatenated to form a comprehensive feature vector of size 43 × 20 = 860 elements, effectively representing each peptide sequence in terms of evolutionary substitution probabilities, while preserving biological significance through biochemical properties and functional relationships refined through evolutionary processes.

PSSM and BLOSUM62 differ fundamentally in their evolutionary information capture approaches. PSSM generates position-specific conservation profiles for individual protein sequences, requiring homologous sequence information and database searches for each target protein, making it computationally intensive but capable of capturing conservation patterns specific to each sequence position. In contrast, BLOSUM62 provides universal substitution probabilities applicable to all protein sequences without requiring sequence-specific homology searches, offering computational efficiency with consistent encoding across all sequences, while capturing general evolutionary substitution patterns across protein families rather than position-specific conservation.

Throughout this work, we use the terms “BLOSUM” and “BLOSUM62” interchangeably to refer to this specific substitution matrix, as BLOSUM62 represents the standard and most widely-used variant of the BLOSUM matrix family for biological sequence analysis.

### Proposed 1DCNN model (BLOS-Khib)

#### Theoretical foundation of CNN and 1DCNN architectures

The CNNs were originally developed by LeCun et al. for image recognition tasks^54^, but have since demonstrated remarkable efficacy in sequence analysis across diverse biological domains^55^. Unlike traditional neural networks that treat input features as independent variables, CNNs exploit spatial and sequential relationships through convolution operations, enabling automatic extraction of hierarchical patterns from structured data. This architectural paradigm addresses the fundamental limitations of fully-connected networks when processing sequential biological data, where local dependencies and positional relationships carry significant functional importance^56^.

The convolution operation represents the core computational mechanism underlying CNN architectures. Given an input sequence and a learnable filter (kernel), convolution computes feature maps by applying the filter across all positions of the input, detecting specific patterns or motifs. This sliding window approach enables parameter sharing across different sequence positions, dramatically reducing the number of trainable parameters while maintaining the network capacity to recognize recurring patterns regardless of their positional occurrence within the sequence^57^.

The 1DCNNs perform convolution operations on flattened sequential data, making them particularly suitable for protein sequence analysis, where amino acid information can be encoded into 1D feature vectors. In proteomics research, protein sequences are typically encoded using various strategies and then flattened into 1D vectors that preserve the essential sequence information while conforming to CNN input requirements. The 1DCNNs have demonstrated effective performance in various computational tasks, including protein function prediction^58^, secondary structure determination^59^, protein-protein interaction analysis^60^, and PTM site identification, by effectively capturing patterns within these flattened sequence representations that correspond to biological motifs and functional signatures^61^.

The hierarchical feature extraction capability of 1DCNNs enables the automatic discovery of patterns within flattened sequence representations without requiring manual feature engineering. Lower convolutional layers typically learn to detect local patterns and correlations within the 1D input vector, while deeper layers combine these elementary features to recognize more complex, longer-range dependencies that span multiple regions of the flattened sequence encoding. This hierarchical representation learning is particularly advantageous for PTM prediction, where modification sites are often characterized by complex signatures that, when flattened, create specific patterns corresponding to amino acid arrangements both upstream and downstream of the target residue.

#### 1DCNN architecture and convolution mechanics

The fundamental operation of 1DCNN involves applying a series of learnable filters across input sequences to generate feature maps that highlight relevant sequence patterns. Given an input sequence $\:X\:\in\:\:\:{\mathbb{R}}^{L\:X_d}$, where $\:L$ is the sequence length and $\:d$ is the feature dimensionality per position, for a filter with kernel $\:W\:\in\:\:\:{\mathbb{R}}^{k\:X_d}\:$ of size $\:k$, the convolution operation at position $\:i$ is computed as:

1 $$y_i = \sum_{j=0}^{k-1} \sum_{f=1}^{d} W_{j,f}\, X_{i+j,f} + b$$

where $\:b$ is the bias term, and $\:{y}_{i}$ is the feature map value at position $\:i$.

For protein sequences encoded using BLOSUM62, where $\:L=43$ and $\:d=20$, the network can effectively process both local sequential patterns and amino acid physicochemical properties, simultaneously. The complete feature map is given as:

2 $$Y = \left( y_{1},\, y_{2},\, \dots,\, y_{L-k+1} \right)$$

It captures the presence and strength of the pattern represented by the filter $\:W$ across all valid positions in the input sequence, integrating information from multiple features at each position.

To detect diverse patterns simultaneously across the input, multiple filters are typically employed in each convolutional layer. For an input vector of flattened dimensionality (e.g., 860), this allows parallel extraction of different types of local features. For a layer with $\:m$ filters, the output consists of $\:m$ feature maps, each highlighting a different aspect of the sequence. The mathematical formulation for the output of the $\:f^{th}$ filter is:

3 $$y^{(f)} = \sigma \Big( X . W^{(f)} + b^{(f)} \Big)$$

where $\:{W}^{\left(f\right)}$ is the filter weight matrix, $\:{b}^{\left(f\right)}$ is the bias, and σ is a non-linear activation function, typically the Rectified Linear Unit (ReLU)^62^.

To reduce dimensionality and retain the most salient features, pooling operations, particularly max pooling, are applied after convolution. Max pooling selects the maximum value within each pooling window along the 1D feature map:

4 $$p_i = \max_{j \in [i \cdot s,\, i \cdot s + w)} Y_j$$

where $\:s$ is the stride, $\:w$ is the pooling window size, and $\:{p}_{i}$ is the pooled value at position $\:i$.

#### Proposed BLOS-Khib architecture and optimization strategy

Based on comprehensive hyperparameter optimization studies conducted using the Python Keras Tuner framework^63^, we developed BLOS-Khib, a specialized 1DCNN architecture optimized specifically for Khib site prediction. The model architecture integrates optimal configurations determined through systematic evaluation of number of filters, kernel size, layer depth, and regularization strategies. The BLOS-Khib architecture consists of the following components arranged in a hierarchical feature extraction pipeline:

##### Input layer

The model accepts BLOSUM62-encoded peptide sequences as 1D vectors of length 860, formed by flattening a 43 × 20 matrix representing each peptide.

##### Hierarchical convolutional architecture

The core architecture consists of six sequential 1D convolutional layers, all using ReLU activation to introduce non-linearity. The initial layers (Conv1–Conv4) depend on a consistent kernel of size 7, progressively refining local features with 256, 384, 320, and 288 filters, respectively. The fifth layer increases feature depth with 512 filters and a reduced kernel size of 3 to focus on fine-grained patterns. The sixth convolutional layer has 128 filters with a kernel size of 5 to consolidate intermediate-scale features before transitioning to the dense layer.

##### Dimensionality reduction

Max pooling with a pool size of 2 is applied after selecting convolutional layers to reduce dimensionality and emphasize dominant features.

##### Regularization strategy

To prevent overfitting, dropout with a rate of 0.1 is applied after the fourth and fifth convolutional layers. Early and final layers are kept dropout-free to preserve initial feature integrity and final consolidation.

##### Feature classification

A fully-connected layer with 384 units integrates extracted features for high-level representation. The final output layer has a sigmoid activation function to generate probabilities indicating the likelihood of Khib modification at the central lysine residue.

##### Training configuration

The model employs the Adam optimizer^64^ with a learning rate of 0.0001 and a batch size of 64, for up to 60 epochs. Early stopping with patience of 10 epochs monitors validation loss to prevent overfitting. Model checkpointing is used to retain the best-performing parameter configuration during training.

##### Loss function

Binary cross-entropy is used as the objective function for distinguishing modified from unmodified lysine sites. Regularization terms are included to encourage generalization and control model complexity.

### Comparative deep-learning architectures

To comprehensively evaluate our proposed BLOS-Khib model, we compared it against several alternative deep-learning architectures that have demonstrated success in sequence-based prediction tasks. This comparison enables us to assess the relative advantages of our CNN-based approach and validate its effectiveness for Khib site prediction across diverse organisms.

The Dense Neural Network (DNN) represents the most fundamental deep learning architecture, consisting of fully-connected layers, where each neuron receives input from all neurons in the previous layer^65^. Unlike architectures designed specifically for sequential data, DNNs process the entire input, simultaneously, treating each position independently without explicitly modelling sequential relationships. Despite this limitation, DNNs provide a useful baseline due to their ability to learn complex nonlinear relationships between input features and target variables.

Recurrent Neural Networks (RNNs) address the sequential nature of protein data by processing amino acids one at a time, while maintaining an internal state that captures information from previously seen residues^66^. LSTM networks^67^, a specialized RNN variant, were designed to overcome the vanishing gradient problem that limits standard RNNs’ ability to capture long-range dependencies. LSTMs incorporate memory cells with input, forget, and output gates that regulate information flow, enabling the network to selectively remember relevant information over extended sequences, a critical capability for identifying patterns in protein data where functional motifs may span multiple residues.

Gated Recurrent Units (GRUs)^68^ serve as streamlined alternatives to LSTMs, combining the forget and input gates into a single “update gate” and merging the cell state with the hidden state. This simplification reduces the number of parameters, while preserving the ability to model long-term dependencies in sequence data. GRUs often achieve performance on par with LSTMs, while offering enhanced computational efficiency, making them an appealing choice for protein sequence analysis.

Bidirectional architectures^69^ enhance standard recurrent models by processing sequences in both forward and reverse directions, simultaneously. This approach allows Bidirectional LSTMs (BiLSTMs) and Bidirectional GRUs (BiGRUs) to incorporate information from both upstream and downstream residues, when making predictions about a central lysine. This bidirectional context is particularly valuable for PTM prediction, as modification sites are typically influenced by amino acid patterns on both the N-terminal and C-terminal sides. By capturing these bidirectional dependencies, BiLSTMs and BiGRUs can potentially identify more complex sequence patterns than their unidirectional counterparts.

Each architecture brings unique strengths to sequence modelling tasks: DNNs offer computational simplicity, LSTMs and GRUs provide mechanisms for capturing long-range sequential patterns, and bidirectional models incorporate comprehensive contextual information from both directions. By comparing our CNN-based approach against this diverse set of architectures, we can rigorously evaluate whether the convolutional operations in BLOS-Khib, which excel at detecting local motifs and position-invariant patterns, offer advantages over alternative sequence modelling strategies for Khib site prediction.

### Evaluation metrics

Comprehensive performance evaluation of computational models for PTM site prediction demands sophisticated assessment methodologies that encompass diverse aspects of classification efficacy. Despite achieving balanced dataset compositions, thorough evaluation protocols remain paramount given the profound biological implications of predictive errors in proteomics research applications. Misclassification events, whether failing to detect actual modification sites (false negatives) or incorrectly identifying unmodified residues as being modified (false positives), carry distinctive consequences for biological understanding and experimental design strategies, thereby requiring diverse complementary performance indicators that examine various facets of the BLOS-Khib predictive framework.

The assessment methodology implemented herein addresses these complexities through a multi-faceted evaluation strategy encompassing: (1) stratified *k*-fold validation protocols^70^ to examine learning proficiency and internal model consistency while preserving class balance across data partitions, (2) holdout test dataset analysis to determine performance on completely novel instances, (3) diverse performance indicators capturing distinct classification characteristics, and (4) comprehensive threshold-independent analysis ensuring reliable comparative evaluation across alternative approaches and encoding methodologies.

Performance quantification depends on six complementary indicators derived from the confusion matrix components: true positives (*TP*), true negatives (*TN*), false positives (*FP*), and false negatives (*FN*):

5 $$\:\begin{array}{c}Accuracy\:\left(ACC\right)=\frac{TP+TN}{TP+TN+FP+FN}\end{array}$$

6 $$\:\begin{array}{c}Sensitivity\:\left(SN\right)=\frac{TP}{TP+FN}\:\end{array}$$

7 $$\:\begin{array}{c}Precision\:\left(PR\right)=\:\frac{TP}{TP+FP}\end{array}$$

8 $$\:\begin{array}{c}F1=\frac{2\times PR\times SN}{\:PR+SN}\end{array}$$

9 $$\:\begin{array}{c}\:MCC=\frac{\left(TP\times TN\right)-\left(FP\times FN\right)}{\sqrt{\left(TP+FP\right)\left(TP+FN\right)\left(TN+FP\right)\left(TN+FN\right)}}\end{array}$$

10 $$\:\begin{array}{c}AUC=\int_{0}^{1}\:TPR\left(FPR^{-1}(x)\right)\:dx\end{array}$$

where *TPR* is the True Positive Rate, and *FPR* is the False Positive Rate.

While accuracy provides a global performance measure, sensitivity focuses on the model capability to detect Khib sites, which is crucial in biological contexts where missing positive instances could impact downstream analyses. Precision assesses the model ability to ensure that predicted Khib sites are true Khib sites, reducing false positives that might lead to misguided biological interpretations. The F1-score balances precision and sensitivity, particularly valuable when both false negatives and false positives carry significant consequences. Matthews Correlation Coefficient (MCC) provides a particularly robust evaluation measure for binary classification tasks with potential class imbalance, considering all four components of the confusion matrix in a single metric.

Performance evaluation was conducted using Receiver Operating Characteristic (ROC) curves, which represent plots of the true positive rate versus the false positive rate across all possible classification thresholds to provide a threshold-independent assessment of model discriminative capability^71^. The AUC was used as the primary metric, with values closer to 1.0 indicating excellent classification performance and values near 0.5 revealing random behaviour. In this study, ROC curves were generated for both cross-validation and independent test evaluations. Cross-validation results were averaged using vertical averaging at fixed false positive rates, with confidence intervals to reflect variability across folds. Independent test sets’ ROC curves provided unbiased estimates of generalization to unseen biological data. We further employed ROC analysis to quantitatively compare feature encoding strategies, model architectures, and classifier types within the BLOS-Khib framework.

## Results

### Comparative sequence signature analysis of Khib sites across species

To investigate the sequence contexts surrounding Khib sites across multiple species, we employed Two-sample sequence logo analysis^72^, a computational method that quantifies and visualizes statistically-significant differences in amino acid composition between two distinct sequence datasets. Unlike conventional sequence logos that display the overall frequency distribution of residues at each position, Two-sample logos specifically highlight positions where amino acid usage differs significantly between positive (Khib-modified) and negative (non-modified) sequence sets.

The Two-sample logo generation process involves several computational steps: alignment of sequences from both positive and negative datasets around the central lysine residue (position 0), calculation of amino acid frequencies at each position for both datasets, statistical testing to identify positions with significant compositional differences using *t*-tests with Bonferroni correction for multiple testing (*P* < 0.05), and visualization of significantly different residues with letter heights proportional to the magnitude of difference and statistical significance. Amino acids appearing above the baseline represent enrichment in Khib-modified sequences relative to non-modified controls, while those below the baseline indicate depletion (reduced frequency) in Khib sites compared to background sequences.

**Fig. 3:**
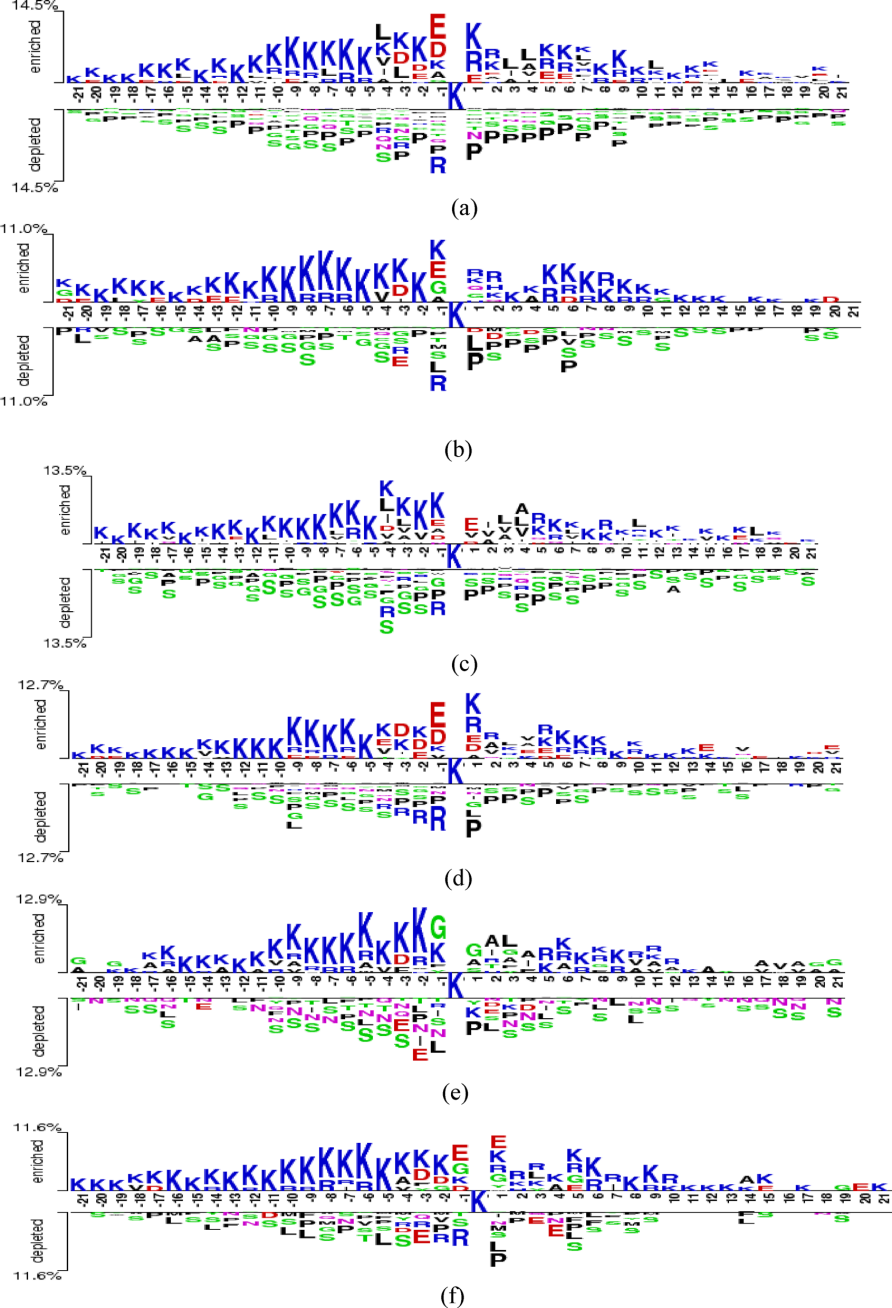
Sequence logos depicting conserved amino acid patterns surrounding Khib sites in (**a**) human, (**b**) wheat, (**c**) *T. gondii*, (**d**) rice, (**e**) Candida, and (**f**) *B. cinerea* datasets. Two-sample logos were generated using Student’s *t*-test with Bonferroni correction (*P* < 0.05).

The *y*-axis percentage values in the logos represent the relative frequency difference between the two sequence sets, quantifying the magnitude of enrichment or depletion for each amino acid at specific positions. This statistical approach ensures that only biologically-meaningful sequence preferences are highlighted, filtering out random compositional variations and focusing on evolutionarily conserved or species-specific modification signatures. The Two-sample sequence logos generated for six species (humans, wheat, *T. gondii*, rice, Candida, and *B. cinerea*) revealed both conserved and species-specific sequence preferences surrounding Khib modification sites (Fig. 3).

In the human sequence logo (Fig. 3a), there is a strong enrichment of lysine (K) across upstream positions − 21 to − 1, generating a positively charged electrostatic environment that likely facilitates the binding of Khib-modifying enzymes. This pattern may reflect structural or functional domains such as histone tails, where Khib was first identified. Notably, glutamic acid (E) is enriched at positions − 2 and − 1, forming a sharp transition to an acidic microenvironment directly preceding the modification site. This contrast between upstream basic and proximal acidic residues may serve as a recognition motif that enhances catalytic specificity or docking efficiency. Additional enrichment of leucine (L) at positions − 3, −4, + 3, and + 4 indicates a preference for hydrophobic residues flanking the core region, possibly contributing to substrate stabilization or secondary structure formation. Downstream regions display pronounced enrichment of arginine (R) at positions + 1, +2, + 5, +6, and + 9, along with additional K residues, revealing continued preference for a high positive charge density post-modification. In contrast, there is a consistent depletion of proline (P) and serine (S) throughout the flanking regions, likely due to their disruptive effects on backbone flexibility or due to steric or chemical incompatibility with modification machinery.

The sequence logo for wheat (Fig. 3b) demonstrates a strong conservation of the human Khib motif. Upstream K enrichment and E enrichment at position − 1 are preserved, establishing a comparable electrostatic recognition environment. The downstream region exhibits enrichment of R and K residues similar to human patterns, reinforcing the importance of positive charge maintenance. As with human sequences, P and S are consistently depleted across flanking positions, supporting the structural requirement for conformational flexibility. In addition, wheat sequences exhibit depletion of alanine (A), glycine (G), and L at various positions, showing a more selective residue profile that excludes small or hydrophobic residues in certain positions to maintain Khib site integrity.

*T. gondii* (Fig. 3c) exhibits sequence features consistent with those observed in mammals and plants. The upstream region contains conserved K enrichment, and E enrichment at position − 1 maintains the key acidic transition zone. The flanking regions also display L enrichment at positions − 3 and − 4, further supporting the conserved use of hydrophobic residues near the modification site. P and S are uniformly depleted across the sequence, reaffirming the structural constraints necessary for modification accessibility. These conserved patterns show that *T. gondii*, despite its parasitic nature, retains the core sequence elements associated with canonical Khib recognition.

The rice logo (Fig. 3d) mirrors the features of other plant and animal systems, including upstream K enrichment and a clear acidic motif with E at position − 1. Downstream R and K residues are also enriched, and P and S are depleted throughout. This consistent sequence composition among cereals and dicots reinforces the hypothesis that the Khib recognition motif is conserved across plant lineages.

The logo for *Candida albicans* (Fig. 3e) reveals a distinct departure from the canonical Khib signature. While K enrichment in upstream regions persists, the typical E enrichment at position − 1 is replaced by G, indicating a unique local sequence environment. This substitution may reflect fungal-specific enzymatic requirements or an alternative substrate recognition strategy. The downstream region is enriched in A and L residues, showing a preference for small or hydrophobic side chains over the positively charged R/K combination found in other organisms. Nevertheless, the depletion of P and S remains consistent with other species, ensuring that structural constraints on Khib accessibility may be universally conserved.

In contrast to Candida, the sequence logo for *Botrytis cinerea* (Fig. 3f) demonstrates a high degree of convergence with canonical Khib signatures. It retains upstream K enrichment and features E enrichment at position − 1, in line with mammalian and plant profiles. The downstream region is similarly enriched in R and K, and the typical P and S depletion is observed throughout. These findings indicate that *B. cinerea*, despite its fungal classification, maintains the conserved molecular recognition framework associated with Khib, unlike the divergence observed in *Candida albicans*.

Taken together, these comparative analyses reveal both conserved and divergent patterns in Khib sequence signatures across species. The enrichment of upstream K and E at position − 1 appears to be a defining feature of Khib site recognition, with implications for enzyme binding and catalytic specificity. The universal depletion of P and S reveals that structural accessibility and conformational flexibility are critical for Khib modification. While most organisms conform to this conserved motif, the divergence observed in *Candida albicans* points to alternative recognition mechanisms that may warrant further experimental investigation. These findings underscore the importance of integrating evolutionary conservation and residue context in computational prediction frameworks for Khib and other lysine modifications.

### Optimization of peptide length for CNN-based Khib site prediction

To develop an accurate prediction model for Khib sites, determining the optimal peptide length (window size) surrounding the target lysine residue is crucial. The window size directly affects the amount of contextual sequence information available to the model and can significantly impact prediction performance. This section describes our systematic approach to identifying the optimal window size for the CNN-based Khib site prediction model.

We implemented a comprehensive optimization strategy using the Keras Tuner library to design and evaluate the CNN architectures across multiple peptide lengths. Window sizes ranging from 35 to 47 amino acids (centred on the target lysine residue) were examined to determine the optimal sequence context for Khib prediction. For this optimization process, we utilized the human dataset as a representative example, with the findings later generalized to the other species datasets.

For each evaluated window size, we conducted extensive hyperparameter optimization to identify the optimal CNN architecture. The hyperparameter search space encompassed: (i) number of convolutional layers (1–6), (ii) number of filters per layer (32–512), (iii) kernel size (3–9), (iv) max-pooling size (2–4), (v) dropout rate (0-0.5), (vi) number of dense layers (1–6), (vii) number of units per dense layer (32–512), (viii) and learning rate (0.1-0.0001).

Model training depended on 10-fold cross-validation to ensure robust performance estimation, with an additional 10% of the data reserved as an independent test set for final evaluation. The optimized CNN architectures for each window size from 35 to 47 amino acids are detailed in Table **S2**. Notable architectural complexity variations were observed across different window sizes, indicating that optimal sequence feature extraction is significantly dependent on peptide length.

The number of convolutional layers varied from 1 (window size 35) to 6 (window sizes 43 and 45), indicating that longer peptide sequences generally required more complex feature extraction pipelines. The configuration with window size 43 depended on 6 convolutional layers with different numbers of filters (128–512) and kernel sizes (3–7), while the smallest window size (35) required only a single convolutional layer with 320 filters and a kernel size of 7.

The number of dense layers also varied across different window sizes, ranging from 1 (window sizes 39 and 43) to 4 (window sizes 45 and 47). This variation reflects the different requirements for feature abstraction and integration, depending on the input sequence length. All models used ReLU activation functions throughout the network and employed max pooling (size = 2) after convolutional layers to reduce spatial dimensions and computational complexity.

Dropout rates were individually optimized for both convolutional and dense layers across all architectures to mitigate overfitting, with values ranging from 0.0 to 0.5. Learning rates were consistently set at either 0.001 or 0.0001, with preference for the lower rate of 0.0001 in most configurations.

A window size of 43 was identified as providing the optimal balance between contextual information and model complexity for Khib site prediction. As demonstrated in Table 3, the 43-amino acid window achieved the highest performance across all evaluation metrics in 10-fold cross-validation with ACC of 0.818, SN of 0.860, PR of 0.794, F1 score of 0.825, and MCC of 0.640. This performance was consistently maintained in the independent test set evaluation, with ACC of 0.823, SN of 0.894, PR of 0.786, F1 score of 0.837, and MCC of 0.653. The consistent results of this window size demonstrate its capacity to provide a sufficient sequence context for capturing relevant biological patterns, while avoiding noise introduction from distally-located residues.

**Fig. 4:**
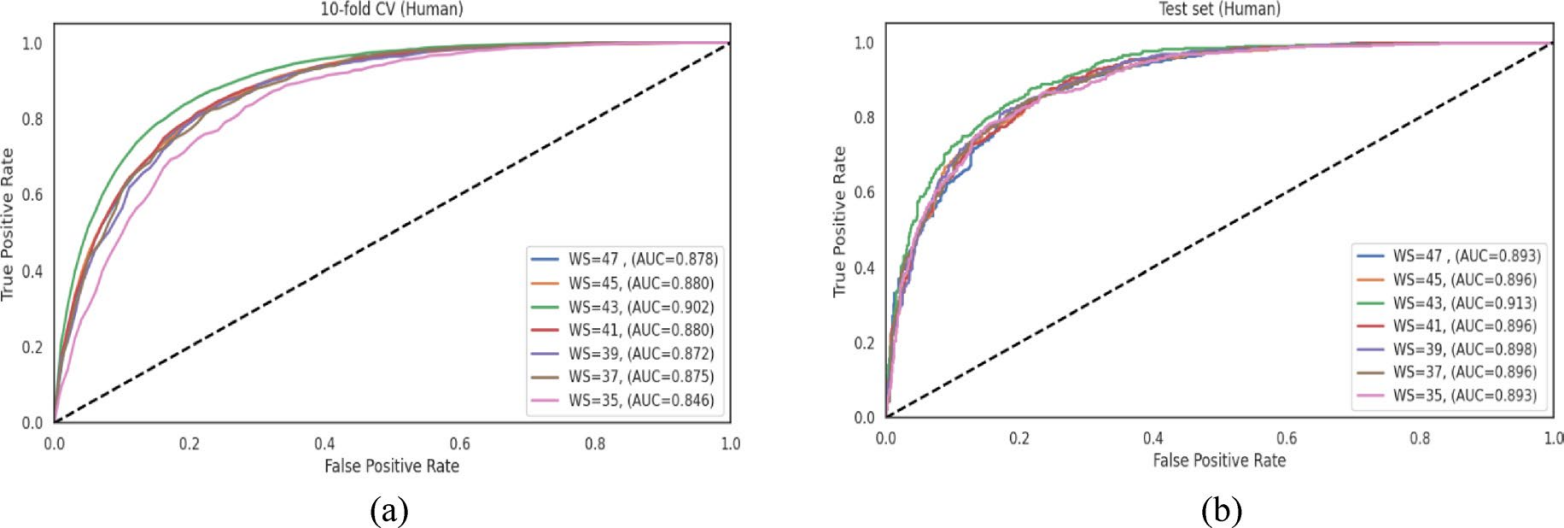
ROC curves comparing the performance of different window sizes (WS) using their respective optimized CNN architectures for Khib site prediction on the human dataset using (**a**) 10-fold cross-validation and (**b**) independent test set.

Figure 4 illustrates ROC curves for different window sizes evaluated on both 10-fold cross-validation and independent test datasets. The 43-amino acid window achieved the highest AUC values of 0.902 and 0.913 for cross-validation and independent test sets, respectively, confirming its effective discriminative capacity for distinguishing between Khib and non-Khib sites relative to alternative window sizes.

The optimized CNN architecture for the 43-amino acid window consisted of 6 convolutional layers and a single dense layer with 384 units, employing minimal dropout regularization and a learning rate of 0.0001. Based on these findings, the 43-amino acid window size was adopted as the standard for developing species-specific Khib prediction models across all six organisms examined in this investigation.

**Table 3:** Performance of different window sizes on the human dataset. Boldface values indicate the best performance for each metric.

Window size	10-fold cross-validation set					Independent test set				
	ACC	SN	PR	F1	MCC	ACC	SN	PR	F1	MCC
47	0.799	0.853	0.769	0.809	0.601	0.809	0.890	0.769	0.825	0.625
45	0.798	0.843	0.773	0.806	0.598	0.800	0.862	0.770	0.814	0.604
**43**	**0.818**	**0.860**	**0.794**	**0.825**	**0.640**	**0.823**	**0.894**	**0.786**	**0.837**	**0.653**
41	0.800	0.848	0.774	0.809	0.604	0.808	0.859	0.783	0.819	0.619
39	0.798	0.846	0.771	0.807	0.598	0.814	0.852	0.795	0.823	0.629
37	0.789	0.851	0.757	0.801	0.583	0.807	0.854	0.783	0.817	0.615
35	0.767	0.802	0.749	0.774	0.535	0.789	0.858	0.758	0.805	0.583

### Assessment of the effect of diverse feature representation methods on the performance of the proposed CNN model

The choice of the feature representation method plays a crucial role on the performance of deep learning models for PTM site prediction. In this study, we evaluated six different feature representation methods: ESM embeddings, one-hot encoding, CTD, PSSM, AAP and BLOSUM62 representation. These methods were assessed across six diverse datasets (humans, wheat, *T. gondii*, rice, Candida, and *B. cinerea*) using our proposed CNN architecture for Khib site prediction.

To optimize predictive performance for each feature representation method, we employed Keras Tuner for hyperparameter optimization of our CNN architecture. The Hyperband algorithm was utilized to efficiently search the same hyperparameter space, tailored specifically for each feature representation method. As detailed in Table **S3**, the optimal CNN architectures varied considerably across feature representation methods.

The BLOSUM62 representation required a deeper architecture with 6 convolution layers, while simpler representations such as ESM and one-hot encoding performed optimally with shallower networks (1 convolution layer each). The dropout rates in convolutional layers progressively decreased from 0.4 for simpler representations (ESM, and one-hot) to 0.0 for the more information-rich BLOSUM62 representation, revealing that the latter model inherent evolutionary information reduces the need for regularization to prevent overfitting. Learning rates were also varied systematically, with 0.001 for ESM, CTD, and PSSM, and 0.0001 for one-hot, AAP, and BLOSUM62, reflecting the need for more precise optimization steps with complex feature representations.

**Fig. 5:**
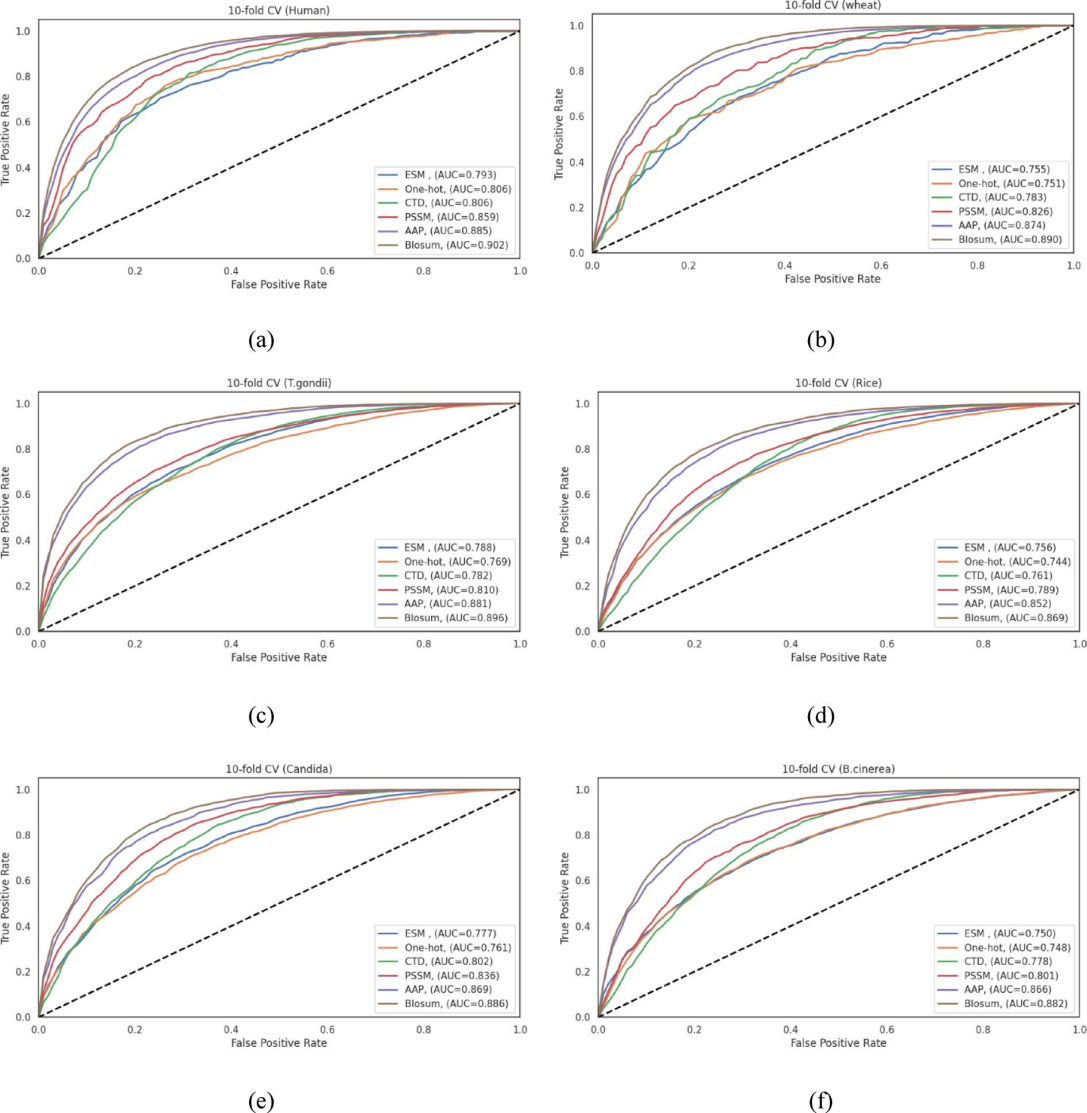
ROC curves for different feature representation methods in Khib site prediction using their respective optimized CNN architectures on the cross-validation sets of the (**a**) human, (**b**) wheat, (**c**) *T. gondii*, (**d**) rice, (**e**) Candida, and (**f**) *B. cinerea* datasets.

Tables 4, 5, 6, 7, 8 and 9 present the comparative performance metrics for all feature representation methods across all datasets, evaluated using both 10-fold cross-validation and a separate 10% independent test set. Four performance metrics were employed: ACC, F1, MCC, and AUC. Figure 5 illustrates the ROC curves for the different feature representation methods across the six datasets during cross-validation, providing a visual comparison of their discriminative capabilities.

The results consistently demonstrate that evolutionary and physicochemical property-based feature representation methods outperform sequence-based and embedding-based methods across all datasets. Specifically, BLOSUM62 exhibited the highest performance in most scenarios, followed closely by AAP. For instance, in the human dataset (Table 4), BLOSUM62 achieved the highest performance with accuracy, F1-score, MCC, and AUC values of 0.818, 0.825, 0.640, and 0.902, respectively, on cross-validation test set, and 0.823, 0.837, 0.653, and 0.913, respectively, on the independent test set. As shown in Fig. 5, the ROC curves for BLOSUM62 and AAP consistently demonstrate larger AUC compared to those of other methods across all datasets, visually confirming their enhanced discriminative capacity. Test set ROC curves showing similar performance trends are available in Fig. S1.

The performance hierarchy across feature representation methods follows a consistent pattern across all datasets: BLOSUM62 > AAP > PSSM > CTD > one-hot > ESM. This trend is evident in the human, wheat, *T. gondii*, rice, Candida, and *B. cinerea* datasets, as shown in Tables 4, 5, 6, 7, 8 and 9. The overall predictive performance varied across datasets, indicating dataset-specific characteristics influencing model efficacy. The human dataset demonstrated the highest overall performance with BLOSUM62, achieving an ACC of 0.823 and an AUC of 0.913 in the test set. The wheat dataset showed comparable but slightly lower performance metrics, with BLOSUM62 yielding an ACC of 0.790 and an AUC of 0.892 in the test set.

Interestingly, the performance gap between feature representation methods varied with dataset. For example, in the human dataset, the difference in accuracy between the best-performing method (BLOSUM62) and the worst-performing method (ESM) was 0.094 in the 10-fold cross-validation, while in the wheat dataset, this difference was 0.123, showing greater sensitivity to feature representation in the latter.

The enhanced performance of BLOSUM62 and AAP can be attributed to their ability to capture evolutionary conservation patterns and physicochemical properties crucial for Khib site prediction. BLOSUM62, being a substitution matrix derived from aligned blocks of protein sequences, effectively encodes evolutionary relationships between amino acids. This evolutionary information appears to be particularly informative for distinguishing Khib modification sites across the diverse datasets examined.

Similarly, AAP, which encapsulates various physicochemical properties of amino acids such as hydrophobicity, polarity, and molecular volume, provides a rich biological context that enhances the model discriminative power. The consistently high performance of AAP across datasets ensures the fundamental importance of physicochemical properties in determining Khib modification sites, regardless of the organism.

The PSSM, which captures position-specific evolutionary information, consistently ranked third in performance, further emphasizing the significance of evolutionary conservation in Khib site prediction. The relatively lower performance of sequence-based methods (one-hot encoding) and embedding-based methods (ESM) shows that simply representing amino acid sequences without incorporating evolutionary or physicochemical context limits the model ability to extract discriminative features specific to Khib modification.

The relatively modest performance of ESM-2 embeddings, despite their demonstrated effectiveness in various protein-related tasks, can be attributed to several factors specific to Khib site prediction. First, ESM-2 embeddings are pre-trained on general protein sequences and may not capture the specific patterns associated with Khib modification sites, which represent a specialized subset of lysine residues. Second, the high-dimensional nature of ESM-2 embeddings with dimensions 43 × 480 may introduce noise when applied to the relatively smaller datasets used in this study, leading to overfitting despite regularization efforts. Third, the contextual information encoded in ESM-2 may be too general compared to the specific evolutionary and physicochemical features captured by BLOSUM62 and AAP, which are more directly relevant to PTM patterns. Finally, the static nature of pre-trained embeddings limits the model ability to adapt representations during supervised training, in contrast to trainable encodings that allow convolutional layers to optimize feature extraction specifically for Khib site discrimination.

This comprehensive evaluation of feature representation methods across six diverse datasets demonstrates that BLOSUM62 and AAP methods consistently achieve higher performance compared to sequence-based and embedding-based approaches when used with our proposed CNN architecture for Khib site prediction. Based on these results, we recommend BLOSUM62 as the optimal feature representation method for Khib site prediction tasks due to its consistently high performance across different organisms and evaluation metrics.

**Table 4:** Performance comparison of feature representation methods using their respective optimized CNN architectures for Khib site prediction in the human dataset. Boldface values indicate the best performance for each metric.

Feature representation method	10-fold cross-validation set				Independent test set			
	ACC	F1	MCC	AUC	ACC	F1	MCC	AUC
ESM	0.724	0.725	0.448	0.793	0.718	0.737	0.439	0.784
One hot	0.745	0.756	0.492	0.806	0.741	0.763	0.487	0.811
CTD	0.741	0.774	0.505	0.806	0.715	0.752	0.445	0.787
PSSM	0.780	0.788	0.561	0.859	0.791	0.809	0.590	0.866
AAP	0.803	0.808	0.608	0.885	0.815	0.822	0.631	0.899
**BLOSUM**	**0.818**	**0.825**	**0.640**	**0.902**	**0.823**	**0.837**	**0.653**	**0.913**

**Table 5:** Performance comparison of feature representation methods using their respective optimized CNN architectures for Khib site prediction in the wheat dataset. Boldface values indicate the best performance for each metric.

Feature representation method	10-fold cross-validation set				Independent test set			
	ACC	F1	MCC	AUC	ACC	F1	MCC	AUC
ESM	0.687	0.707	0.377	0.755	0.697	0.704	0.394	0.754
One hot	0.693	0.683	0.387	0.751	0.667	0.662	0.335	0.742
CTD	0.708	0.743	0.434	0.783	0.744	0.777	0.505	0.807
PSSM	0.745	0.763	0.496	0.826	0.754	0.761	0.508	0.825
AAP	0.798	0.806	0.598	0.874	0.791	0.800	0.583	0.866
**BLOSUM**	**0.810**	**0.817**	**0.626**	**0.890**	**0.790**	**0.777**	**0.586**	**0.892**

**Table 6:** Performance comparison of feature representation methods using their respective optimized CNN architectures for Khib site prediction in the *T. gondii* dataset. Boldface values indicate the best performance for each metric.

Feature representation method	10-fold cross-validation set				Independent test set			
	ACC	F1	MCC	AUC	ACC	F1	MCC	AUC
ESM	0.711	0.713	0.423	0.788	0.711	0.727	0.429	0.800
One hot	0.695	0.694	0.392	0.769	0.705	0.699	0.409	0.778
CTD	0.712	0.734	0.430	0.782	0.720	0.727	0.442	0.778
PSSM	0.731	0.733	0.462	0.810	0.747	0.738	0.493	0.820
AAP	0.800	0.804	0.601	0.881	0.800	0.793	0.600	0.881
**BLOSUM**	**0.815**	**0.822**	**0.634**	**0.896**	**0.804**	**0.800**	**0.609**	**0.893**

**Table 7:** Performance comparison of feature representation methods using their respective optimized CNN architectures for Khib site prediction in the rice dataset. Boldface values indicate the best performance for each metric.

Feature representation method	10-fold cross-validation set				Independent test set			
	ACC	F1	MCC	AUC	ACC	F1	MCC	AUC
ESM	0.690	0.699	0.380	0.756	0.676	0.687	0.352	0.745
One hot	0.682	0.682	0.365	0.744	0.670	0.684	0.338	0.733
CTD	0.703	0.735	0.421	0.761	0.708	0.751	0.425	0.762
PSSM	0.725	0.736	0.453	0.789	0.733	0.746	0.464	0.807
AAP	0.777	0.785	0.556	0.852	0.764	0.771	0.529	0.852
**BLOSUM**	**0.785**	**0.794**	**0.577**	**0.869**	**0.807**	**0.822**	**0.614**	**0.887**

**Table 8:** Performance comparison of feature representation techniques using their respective optimized CNN architectures for Khib site prediction in the Candida dataset. Boldface values indicate the best performance for each metric.

Feature representation method	10-fold cross-validation set				Independent test set			
	ACC	F1	MCC	AUC	ACC	F1	MCC	AUC
ESM	0.706	0.722	0.416	0.777	0.676	0.722	0.426	0.771
One hot	0.693	0.695	0.387	0.761	0.670	0.705	0.408	0.775
CTD	0.735	0.760	0.483	0.802	0.708	0.766	0.482	0.806
PSSM	0.759	0.773	0.524	0.836	0.733	0.752	0.494	0.837
AAP	0.788	0.796	0.578	0.869	0.796	0.807	0.593	0.869
**BLOSUM**	**0.804**	**0.809**	**0.611**	**0.886**	**0.801**	**0.803**	**0.602**	**0.885**

**Table 9:** Performance comparison of feature representation methods using their respective optimized CNN architectures for Khib site prediction in the *B. cinerea* dataset. Boldface values indicate the best performance for each metric.

Feature representation method	10-fold cross-validation set				Independent test set			
	ACC	F1	MCC	AUC	ACC	F1	MCC	AUC
ESM	0.706	0.635	0.368	0.750	0.676	0.691	0.373	0.748
One hot	0.693	0.681	0.370	0.748	0.670	0.667	0.324	0.737
CTD	0.735	0.740	0.443	0.778	0.708	0.767	0.465	0.794
PSSM	0.759	0.739	0.461	0.801	0.733	0.754	0.459	0.803
AAP	0.790	0.796	0.583	0.866	0.791	0.804	0.583	0.865
**BLOSUM**	**0.800**	**0.806**	**0.604**	**0.882**	**0.819**	**0.833**	**0.635**	**0.903**

### Comparative analysis of feature fusion methods for enhancing CNN model performance

A series of feature fusion experiments combining BLOSUM62 matrix representation with other feature extraction methods were conducted, as recommended by numerous studies in the PTM prediction field^41,42,73^. Five different feature fusion methods were evaluated across six diverse datasets (humans, wheat, *T. gondii*, rice, Candida, and *B. cinerea*).

We optimized CNN architectures for each feature fusion method using Keras Tuner with the Hyperband algorithm, employing the same hyperparameter search space defined above . As detailed in Table **S4**, optimal architectures varied considerably across fusion methods. BLOSUM + AAP required 6 convolutional layers, while BLOSUM + ESM needed only 2. Filter sizes, max pooling strategies (pools of 2–4), dropout rates, and dense layer configurations (1–4 layers) also varied significantly across fusion methods. Most fusion methods performed optimally with a 0.0001 learning rate, except BLOSUM + CTD and BLOSUM + PSSM, which required a rate of 0.001.

Tables **S5-S10** present the comprehensive performance metrics of the five different feature fusion methods across all six datasets. Our results reveal that fusion methods did not substantially enhance performance compared to the standalone BLOSUM representation identified as the highest-performing method in our previous experiments, as revealed in Tables 4–9. For instance, in the human dataset, as illustrated in Table **S5**, the best fusion method is BLOSUM + ESM. It achieved an ACC of 0.814, an MCC of 0.627, and an AUC of 0.897 on the test set, which are comparable to the results obtained using BLOSUM alone.

This pattern was consistent across all datasets examined. In the wheat dataset, as revealed in Table **S6**, while BLOSUM + PSSM showed the highest cross-validation accuracy of 0.807, this performance was not substantially higher than that of BLOSUM in isolation, which achieved an ACC of 0.810, an F1-score of 0.817, and an MCC of 0.626 in cross-validation, as shown in Table 6. When examining discrimination capability, standalone BLOSUM achieved an AUC of 0.892 on the wheat test set, compared to BLOSUM + PSSM fusion, which achieved an AUC of 0.855.

**Fig. 6:**
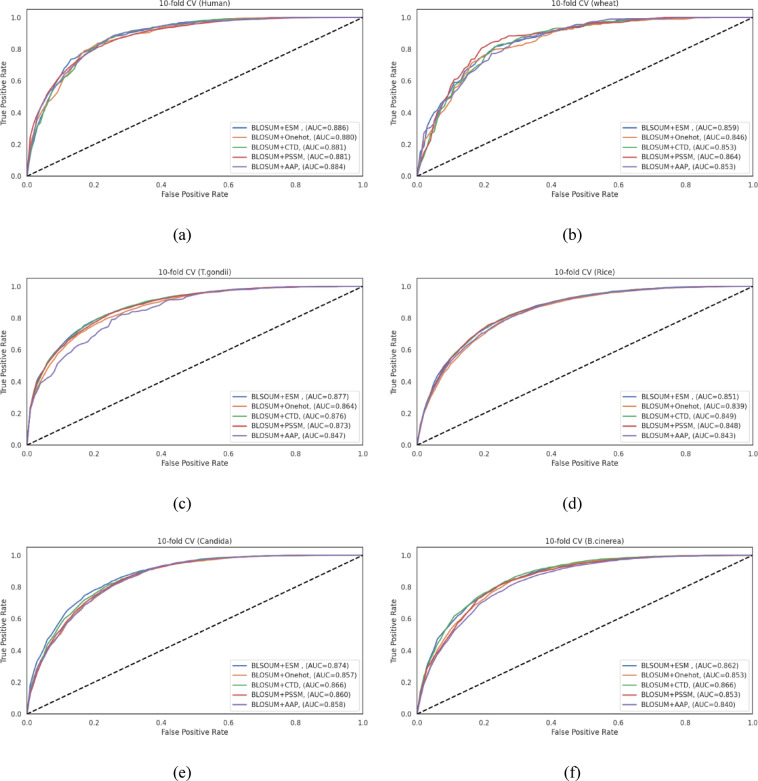
ROC curves demonstrating the discriminative performance of various feature fusion strategies for Khib site prediction using their respective optimized CNN architectures across cross-validation sets of the (**a**) human, (**b**) wheat, (**c**) *T. gondii*, (**d**) rice, (**e**) Candida, and (**f**) *B. cinerea* datasets.

Similarly, for *T. gondii*, rice, Candida, and *B. cinerea* datasets, as shown in Tables **S7-S10**, none of the fusion methods demonstrated marked improvement over the standalone BLOSUM representation. Figure 6 illustrates the ROC curves for the 10-fold cross-validation results across all six datasets, showing the discriminative capability of the different feature fusion methods. The corresponding ROC curves for the independent test sets are provided in Fig. **S2** for comprehensive evaluation.

This finding might be attributed to several factors: (1) the inherently strong representational capacity of BLOSUM matrices for capturing evolutionary information relevant to Khib sites; (2) the potential redundancy or noise introduced when combining features with overlapping information content; and (3) the specific architecture of our CNN model, which appears to efficiently extract discriminative patterns from BLOSUM representations without requiring additional feature types.

Based on these comprehensive analyses of individual feature representation methods and their fusion combinations, we established our proposed CNN model with BLOSUM encoding as our final architecture, hereafter referred to as BLOS-Khib. All subsequent analyses in this study depend on BLOSUM as the feature representation method.

### Comparative analysis of alternative deep learning architectures for Khib prediction

To validate the effectiveness of our CNN-based approach, we conducted a comprehensive evaluation examining the performance of various deep learning architectures, including DNN, LSTM, GRU, BiLSTM, and BiGRU as alternatives to our proposed CNN-based BLOS-Khib model across six diverse datasets. This comparative analysis aimed to determine whether alternative architectures could achieve comparable or enhanced performance for Khib site prediction.

All models were systematically optimized using Keras Tuner with the hyperparameter search methodology described above to ensure a fair comparison. As detailed in Table **S11**, the optimal configurations varied significantly between model types. The BiGRU model required only a single layer with 96 neurons, while the optimal LSTM configurations demanded three layers (256, 32, and 416 neurons). These architectural differences show that different model types extract sequence features through distinct mechanisms, with varying complexity requirements.

The optimization process revealed that bidirectional architectures, namely BiLSTM and BiGRU, generally required fewer layers than those of their unidirectional counterparts, indicating that bidirectional processing of sequence information may provide more efficient feature extraction. Traditional DNN achieved optimal performance with simpler single-layer architectures, while recurrent networks benefited from deeper configurations.

**Fig. 7:**
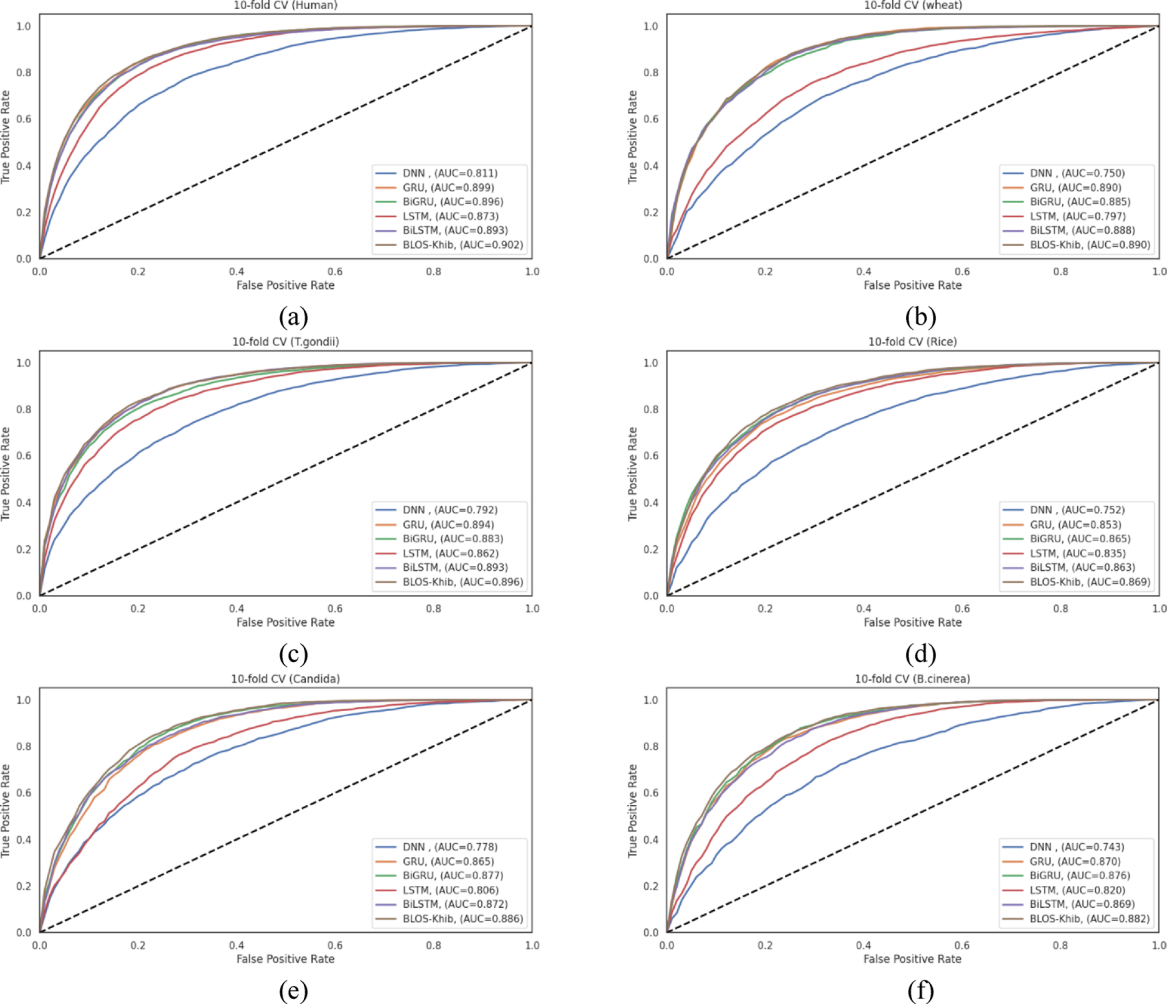
ROC curves comparing optimized deep learning models with BLOS-Khib on cross-validation sets from six datasets: (**a**) human, (**b**) wheat, (**c**) *T. gondii*, (**d**) rice, (**e**) Candida, and (**f**) *B. cinerea*.

Tables **S12-S17** present detailed performance metrics for each model configuration across the six species datasets, evaluated using both 10-fold cross-validation and independent test sets. Figure 7 presents the ROC curves for cross-validation sets, while Figure **S3** provides the corresponding independent test set ROC curves, visually illustrating the discriminative capabilities of different architectures. The BLOS-Khib model achieved the highest MCC values during both cross-validation (ranging from 0.577 for rice to 0.640 for human datasets) and indepenent testing (ranging from 0.586 for wheat to 0.653 for human datasets), demonstrating balanced performance in identifying both positive and negative samples.

The bidirectional recurrent networks showed consistently high performance across all evaluation metrics, with BiGRU particularly demonstrating robust discriminative capability. The performance gap between CNN and bidirectional recurrent architectures was relatively modest, revealing that both approaches effectively capture sequential patterns relevant to Khib site prediction.

The enhanced performance of CNN and bidirectional recurrent architectures can be attributed to their ability to capture different aspects of sequence information. CNNs excel at detecting local patterns and motifs through convolutional operations, while bidirectional recurrent networks effectively model long-range dependencies by processing sequences in both forward and backward directions. The modest performance of unidirectional recurrent networks (LSTM and GRU) compared to their bidirectional counterparts shows that context from both directions of the sequence is important for accurate Khib site prediction. The relatively poor performance of traditional DNN indicates that simply processing flattened sequence representations without considering sequential relationships limits discriminative capability.

These results support the selection of our CNN-based approach with BLOSUM encoding for Khib site prediction, as it consistently achieved the highest performance across diverse organisms. The strong performance of bidirectional recurrent networks, particularly BiGRU, ensures that combining convolutional and recurrent elements could represent a promising direction for future research in PTM prediction.

### Comparison of BLOS-Khib with traditional machine learning classifiers

To comprehensively evaluate the efficacy of our proposed CNN-based BLOS-Khib model, we conducted a comparative analysis against well-established machine learning classifiers, including K-Nearest Neighbours (KNN)^74^, SVM^75^, Random Forest (RF)^76^, Extreme Gradient Boosting (XGBoost)^77^, Light Gradient Boosting (LightGBM)^78^, and Categorical Boosting (CatBoost)^79^. This comparison aimed to assess whether traditional machine learning approaches could achieve comparable performance to those of deep learning methods for Khib site prediction.

The machine learning classifiers underwent rigorous hyperparameter optimization to ensure a fair comparison with our deep learning approach. KNN implemented Euclidean distance metrics with 9 neighbours, while RF utilized 500 decision trees with Gini impurity for split decisions. SVM employed a polynomial kernel for nonlinear classification. The gradient boosting methods received particular attention: XGBoost operated with a 0.1 learning rate, 15-level tree depth, 500 estimators, and combined *L*_1_/*L*_2_ regularization (*λ* = 1 and *α* = 2); CatBoost ran for 500 iterations with default settings; LightGBM was extensively tuned, with optimal parameters including 500 estimators, 0.1 learning rate, maximum depth of 11, and 89 leaves.

Tables **S18-S23** present the performance metrics across the six diverse datasets, evaluated using both 10-fold cross-validation and independent test sets. Among traditional machine learning approaches, ensemble-based methods (XGBoost, LightGBM, and CatBoost) achieved higher performance compared to KNN, SVM, and RF. LightGBM generally emerged as the most competitive traditional classifier, particularly for the human and Candida datasets. However, even the best-performing traditional classifier achieved lower performance than that of our CNN-based approach, with AUC improvements of 4.1–6.5% during cross-validation and 3.4–7.2% on independent test sets when comparing BLOS-Khib to the next best method.

The performance differences were particularly pronounced for the rice and *B. cinerea* datasets as shown in Tables **S21 and S23**. BLOS-Khib achieved higher performance than that of the best traditional classifier by margins of 6.2% and 7.0% in AUC values on independent test sets, respectively. The KNN algorithm consistently demonstrated the lowest performance across all datasets, with AUC values approximately 25–30% lower than those of our proposed model. The performance hierarchy among traditional classifiers remained relatively consistent across datasets: LightGBM > CatBoost > XGBoost > Random Forest > SVM > KNN. This pattern ensures that ensemble methods, particularly gradient boosting ones, are more effective for capturing the complex patterns associated with Khib site prediction compared to simpler algorithms.

**Fig. 8:**
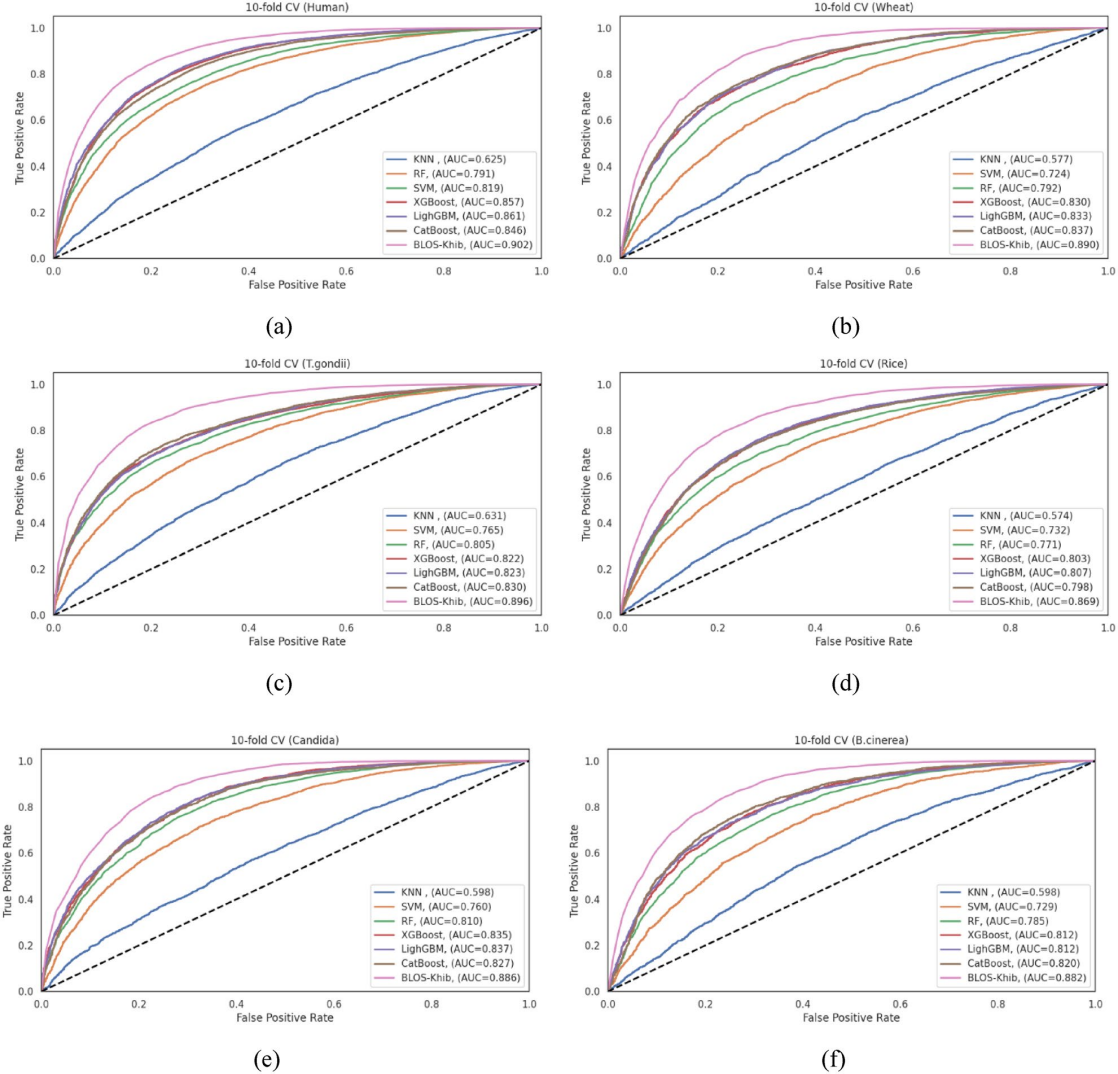
ROC curves showing a comparison of machine learning classifiers with BLOS-Khib on cross-validation sets from six datasets: (**a**) human, (**b**) wheat, (**c**) *T. gondii*, (d) rice, (**e**) Candida, and (**f**) *B. cinerea*.

Figure 8 provides visual confirmation of these findings, clearly illustrating the enhanced discriminative capability of BLOS-Khib compared to all traditional machine learning approaches during cross-validation. The ROC curves demonstrate that our CNN-based model achieves consistently larger areas under the curve across all six datasets. Complementary ROC curves for test set performance are provided in Figure **S4**, which further validates these observations.

### Cross-species applicability of the BLOS-Khib model

#### General model performance across multiple species

**Fig. 9:**
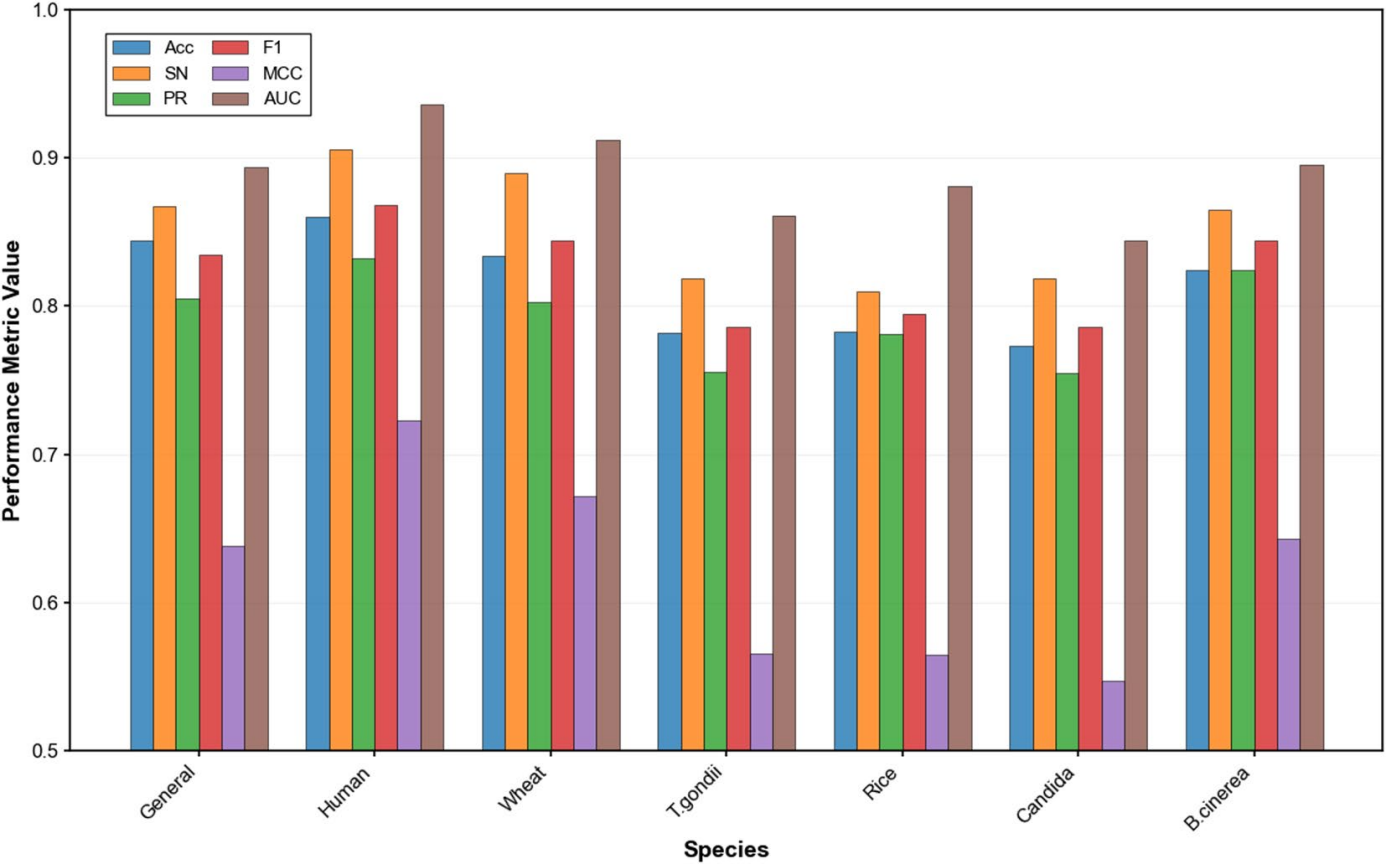
Comparative analysis of the BLOS-Khib general model across different species.

To investigate the cross-species applicability of our CNN-based BLOS-Khib approach, we created a general model by merging all species-specific datasets into a consolidated training set. This general model was systematically evaluated on the test sets from each species dataset, and on a general test set comprising samples from all species. Figure 9 presents the comprehensive performance metrics for these evaluations.

The general BLOS-Khib model demonstrated robust cross-species predictive capabilities with varying degrees of effectiveness across different taxonomic groups. The model achieved the highest performance on the human test set (ACC = 0.860, AUC = 0.936, MCC = 0.723), followed by wheat (ACC = 0.834, AUC = 0.912) and *B. cinerea* (ACC = 0.824, AUC = 0.895). In comparison, performance was moderately lower on *T. gondii* (ACC = 0.782, AUC = 0.861), rice (ACC = 0.783, AUC = 0.881), and Candida (ACC = 0.773, AUC = 0.844) test sets.

Notably, the model consistently maintained high SN values across all species, ranging from 0.810 to 0.906, indicating reliable detection of positive cases regardless of the target organism. The PR values displayed somewhat greater variability, from 0.755 to 0.832, showing that the model ability to correctly identify negative cases is more dependent on the species. The MCC, which provides a balanced measure of classification performance, revealed the strongest correlation for human (0.723) and wheat (0.672) predictions, with somewhat lower values for the remaining species, ranging from 0.547 to 0.643.

#### Cross-species transferability of species-specific models

**Fig. 10:**
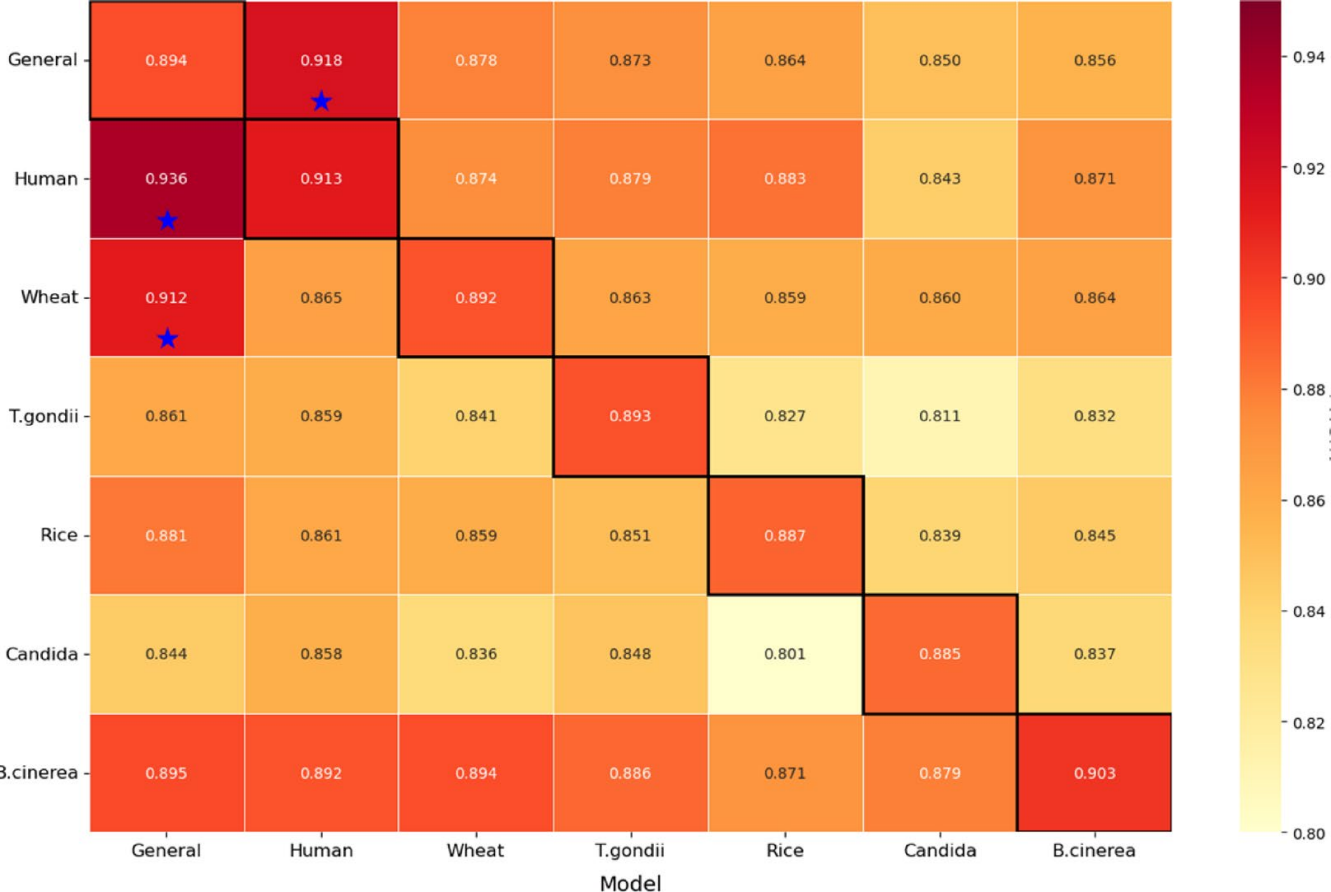
Heatmap showing the AUC values of different BLOS-Khib models (columns) on various test datasets (rows). Diagonal cells (black borders) represent species-specific performance. Blue stars (★) indicate cases where a non-native model outperforms the native model.

To further explore the transferability of localization prediction across species boundaries, we conducted comprehensive cross-testing experiments. Each species-specific BLOS-Khib model was evaluated against the test sets from all other species, with AUC values from these evaluations presented in Fig. 10. Analysis of the cross-species applicability revealed several significant patterns:

- The wheat model demonstrated notably high performance on the *B. cinerea* test dataset with an AUC of 0.894, approaching the performance of the *B. cinerea* model on its native test data with an AUC of  0.903. This shows potential evolutionary conservation of Khib site features between these taxonomically distant organisms or convergent adaptation of modification mechanisms.
- The general model achieved high transferability to the human test set with an AUC of 0.936, compared to the general model performance on its comprehensive test data with an AUC of 0.894. This indicates that features learned from diverse species datasets are highly applicable to human Khib site prediction.
- The human-trained model showed strong performance on the general test set with an AUC of  0.918, comparable to the human-specific model performance with an AUC of  0.913. This reciprocal relationship between human and general models indicates that human Khib site features are well-represented in the consolidated dataset.
- The human model performed well on the rice test dataset with an AUC of 0.861, while the rice model showed strong performance on the human test dataset with an AUC of  0.883, revealing substantial cross-species applicability between these evolutionarily distant organisms.
- *T. gondii*, rice, and Candida test sets achieved optimal prediction when evaluated by their respective species-specific models with AUC values of 0.893, 0.887, and 0.885, respectively, indicating that these organisms may possess more distinct Khib site features that benefit from species-specific training.

These findings collectively indicate that while species-specific models generally perform well on their native test data, significant cross-species transferability exists, particularly between certain evolutionary lineages. The exceptional performance of the wheat model on *B. cinerea* data and the general model on human data challenges the conventional assumption that species-specific training always yields optimal results. These patterns indicate that strategic model selection based on cross-species applicability could enhance prediction accuracy in scenarios in which training data for target organisms is limited.

### Comparative performance analysis of BLOS-Khib against existing predictors

To contextualize the performance of our proposed BLOS-Khib model within the current landscape of Khib site prediction tools, we conducted a comprehensive comparative analysis against four state-of-the-art predictors: iLys-Khib, KhibPred, DeepKhib, and ResNetKhib. To ensure methodological rigour and comparative validity, we reimplemented each model according to its original specifications and trained it on our curated datasets under identical experimental conditions.

The existing tools represent a progression in methodological sophistication for Khib site prediction. iLys-Khib and KhibPred employ traditional machine learning approaches with engineered feature sets, while DeepKhib and ResNetKhib leverage deep learning architectures that automatically extract features from sequence data.

The iLys-Khib depends on a 35-residue window centred on the lysine and has a fuzzy SVM to mitigate dataset noise by assigning variable weights to samples based on their relevance and proximity to the class center. The model incorporates three feature encoding methods: Amino Acid Factors (AAF), Binary Encoding (BE), and Composition of k-spaced Amino Acid Pairs (CKSAAP). Feature selection is performed using the Maximum Relevance Minimum Redundancy (mRMR) method to retain the most informative features.

The KhibPred similarly employs AAF, BE, and CKSAAP encoding techniques but with a narrower 29-residue window. It addresses class imbalance through an ensemble SVM classifier approach, where negative samples are divided into seven subsets, with individual SVMs trained on each subset combined with the positive samples. The final prediction aggregates outputs from all SVMs in the ensemble.

The DeepKhib represents a methodological advancement employing a deep learning framework with CNN architecture and a one-hot encoding approach. The model has a four-layer architecture comprising: (i) an input layer with one-hot encoding representation, (ii) a convolution layer containing four convolution sublayers with 128 filters of lengths 1, 3, 9, and 10, along with two max pooling sublayers, (iii) a fully-connected layer incorporating global average pooling to prevent overfitting, and (iv) an output layer with sigmoid activation function for probability scoring. The model depends on a 37-residue window for sequence analysis.

The ResNetKhib advances the deep learning approach as the first cell-type-specific deep learning predictor for lysine Khib sites. It depends on a residual neural network (ResNet) architecture with one-dimensional convolution and transfer learning strategies across different cell types and species. The model architecture comprises five key components: (i) an input layer, (ii) an embedding layer, (iii) a convolution module containing six blocks with residual connections, including a first block with 64 filters followed by five residual blocks, (iv) a fully-connected layer with 16 neurons for feature flattening, and (v) an output layer with sigmoid activation for probability scoring. Like DeepKhib, ResNetKhib has a 37-residue window for sequence context.

**Fig. 11:**
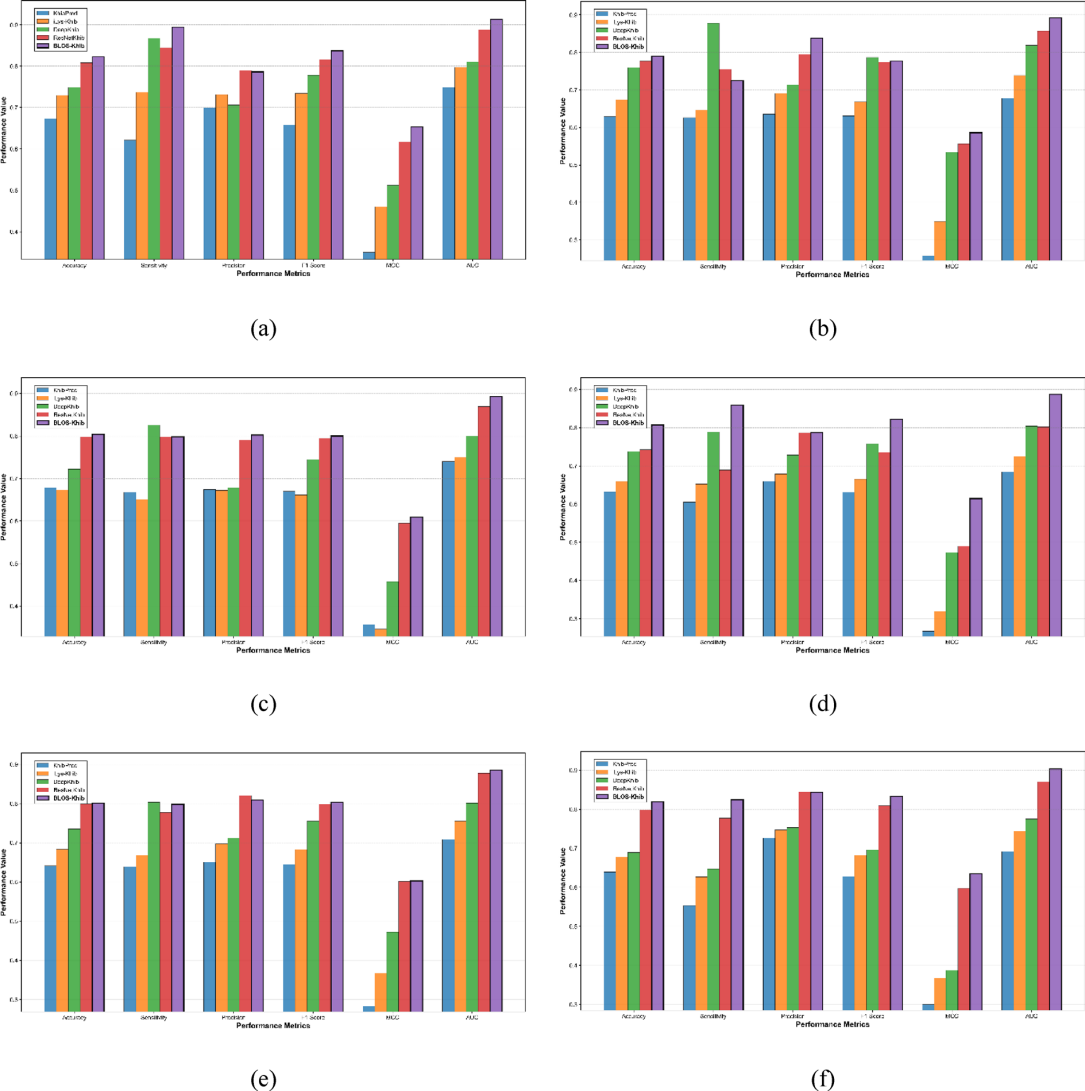
Comparative analysis of KhibPred, iLys-Khib, DeepKhib, ResNetKhib, and BLOS-Khib predictors across all evaluation metrics for (**a**) human, (**b**) wheat, (**c**) *T. gondii*, (**d**) rice, (**e**) Candida, and (**f**) *B. cinerea* test datasets.

Figure 11 provides a comprehensive performance comparison of all models across six species datasets. The results demonstrate that BLOS-Khib consistently outperforms existing predictors across all evaluation metrics and all species datasets.

For the human dataset as shown in Fig. 11a, KhibPred showed modest performance with an accuracy of 0.673 and an MCC of 0.350, while iLys-Khib demonstrated incremental improvement with ACC of  0.730 and MCC of 0.460. DeepKhib achieved better results with an accuracy of 0.749 and an MCC of 0.512, particularly excelling in SN with a value of 0.867 but showing lower PR of 0.706. ResNetKhib emerged as the second-best performer with an accuracy of 0.808 and an MCC of 0.617, demonstrating a more balanced SN of 0.845 and PR of 0.790. BLOS-Khib surpassed all methods with the highest accuracy of 0.823 and MCC of 0.653.

In the wheat dataset (Fig. 11b), traditional machine learning approaches (KhibPred and iLys-Khib) showed limited effectiveness with accuracy values of 0.629 and 0.674, and low MCC scores of 0.258 and 0.349, respectively. DeepKhib demonstrated substantial improvement with accuracy of 0.759 and MCC of 0.533, particularly excelling with SN of 0.878, while sacrificing PR with a value of 0.713. ResNetKhib achieved a better balanced performance with higher PR of 0.795, ACC of 0.777, and MCC of 0.556. BLOS-Khib achieved the highest performance with ACC of 0.790 and MCC of 0.586.

On the *T. gondii* dataset (Fig. 11c), ResNetKhib (ACC of  0.798 and MCC of 0.595) achieved notably higher performance compared to both DeepKhib (ACC of 0.722 and MCC of  0.457) and the traditional machine learning approaches (KhibPred: ACC of 0.678 and MCC of 0.356; iLys-Khib: ACC of  0.673 and MCC of 0.346). Notably, ResNetKhib achieved balanced SN and PR with values of 0.798 and 0.791, respectively, indicating good generalization. BLOS-Khib showed further improvement with the highest ACC of 0.804 and MCC of 0.609.

The rice dataset as shown in Fig. 11d revealed substantial performance differences between deep learning and traditional approaches. DeepKhib achieved an accuracy of 0.737 and an MCC of 0.473, with a good SN of 0.788 but with a lower PR of 0.728. ResNetKhib showed comparable overall performance with an ACC of  0.742 and an MCC of 0.489, but with a different balance, favouring PR with a value of 0.787 and SN with a value of 0.689. In contrast, KhibPred and iLys-Khib showed markedly lower accuracy values of 0.632 and 0.659 and MCC values of 0.267 and 0.319, resppectively. BLOS-Khib achieved the highest performance with an ACC of 0.807 and an MCC of 0.614.

For the Candida dataset as shown Fig. 11e, ResNetKhib demonstrated good performance with an accuracy of 0.799 and an MCC of 0.601, particularly excelling in PR with a value of 0.820. DeepKhib achieved moderate results with an ACC of 0.735 and an MCC of 0.472, while traditional approaches again lagged significantly (KhibPred: ACC of 0.641and MCC of 0.283; and iLys-Khib: ACC of 0.683 and MCC of 0.367). BLOS-Khib achieved slightly higher performance than that of ResNetKhib with an ACC of 0.801 and an MCC of 0.602.

The *B. cinerea* dataset, as shown in Fig. 11f, exhibited the most pronounced performance gradient across models. ResNetKhib achieved an ACC of 0.798 and an MCC of 0.597, significantly outperforming DeepKhib with an ACC of  0.689 and an MCC of 0.387; iLys-Khib with an ACC of 0.678 and an MCC of 0.367; and KhibPred with an ACC of  0.639 and an MCC of 0.300. ResNetKhib demonstrated a particularly good PR of 0.844, indicating effective discrimination of non-Khib sites. BLOS-Khib achieved the best overall performance with an ACC of 0.819 and an MCC of 0.635.

**Fig. 12:**
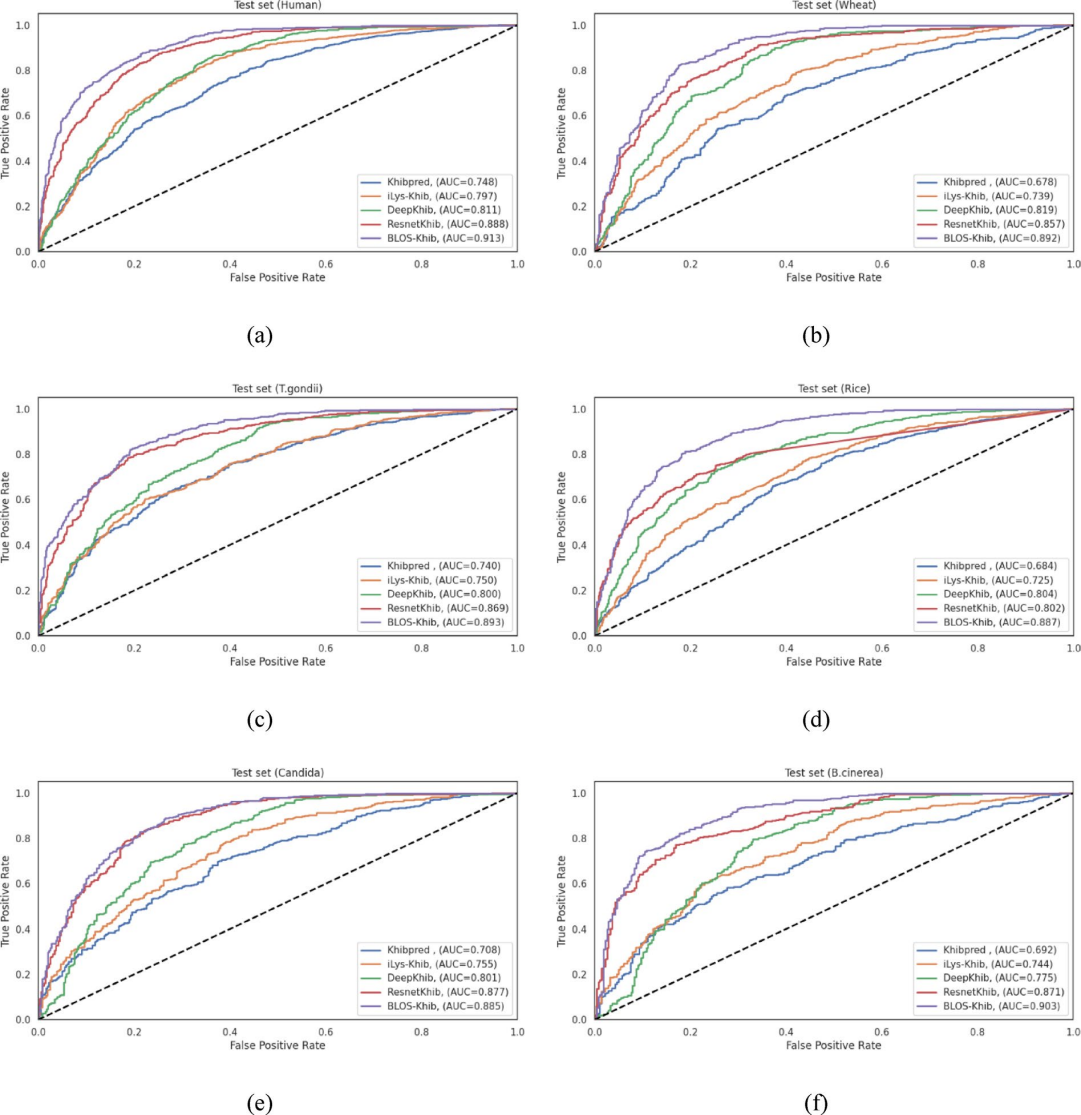
ROC curves comparing existing methods for predicting Khib with BLOS-Khib on test sets from six datasets: (**a**) human, (**b**) wheat, (**c**) *T. gondii*, (**d**) rice, (**e**) Candida, and (**f**) *B. cinerea*.

The ROC curves presented in Fig. 12 visually confirm the performance advantages of the proposed BLOS-Khib model over the existing methods across all species datasets, with AUC values following the same trends as those of the accuracy and MCC metrics discussed above.

The performance differential between BLOS-Khib and ResNetKhib, the second-best performer, is particularly informative. While both leverage deep learning architectures (1DCNN and ResNet, respectively), BLOS-Khib integration of BLOSUM-encoded features appears to provide additional discriminative power for identifying Khib sites. This ensures that pure sequence-based approaches using one-hot encoding, even with sophisticated architectures like residual networks, may benefit from incorporating evolutionary substitution patterns and biochemical knowledge embodied in the BLOSUM matrix.

The comparative analysis also reveals progressive performance improvements corresponding to methodological sophistication: from traditional SVM (KhibPred and iLys-Khib) to CNN (DeepKhib) to residual networks (ResNetKhib) to BLOSUM-encoded 1DCNN (BLOS-Khib). This pattern underscores the value of not only architectural innovations but also feature representation strategies in advancing the field of PTM prediction.

## Conclusion

This study introduced BLOS-Khib, a deep learning framework that leverages BLOSUM62-encoded evolutionary information within a convolutional neural network to predict Khib sites across taxonomically diverse organisms. Through systematic optimization, we established that a 43-residue window effectively captures the sequence context essential for Khib prediction, while comprehensive comparative analyses demonstrated the effectiveness of evolutionary-based representations over alternative encoding strategies. BLOS-Khib consistently achieved higher performance compared to those of existing predictors and alternative deep learning architectures across all datasets, with AUC values ranging from 0.885 to 0.913. The model exhibited notable cross-species transferability, particularly between evolutionarily distant organisms such as wheat and *B. cinerea*, showing the conservation of fundamental Khib recognition patterns. However, several limitations should be acknowledged. These include the absence of three-dimensional structural information that could provide spatial context beyond the primary sequence and the lack of consideration for crosstalk with other PTMs that may influence Khib site selection. Additionally, the use of artificially balanced datasets may not reflect natural class distributions, and experimental validation of predicted sites remains to be performed. Finally, the evaluation of higher-capacity ESM variants was limited by computational resource constraints.

The practical applications of BLOS-Khib extend across multiple domains of biological research and biotechnology. In basic research, the predictor enables large-scale identification of potential Khib sites in newly sequenced genomes, facilitating functional annotation and comparative genomic studies. For drug discovery and therapeutic development, BLOS-Khib can assist in identifying key regulatory sites that may serve as targets for pharmacological intervention, particularly in diseases where Khib dysregulation plays a role. In agricultural biotechnology, the tool cross-species capabilities make it valuable for crop improvement programs, enabling the identification of regulatory modifications that influence stress resistance, yield, or nutritional content. Additionally, the predictor supports protein engineering efforts by helping researchers understand which lysine residues are likely to undergo Khib modification, thereby informing rational design strategies. Future research directions should explore multi-label approaches capable of simultaneously predicting multiple PTMs to account for regulatory crosstalk, integration of structural information, including protein tertiary structure and solvent accessibility metrics, and continued refinement of species-specific models as additional Khib data becomes available. Overall, BLOS-Khib offers a methodological advancement in computational PTM prediction, yielding valuable insights into the sequence determinants and evolutionary conservation of Khib, and setting the stage for broader biological discovery and translational applications.

## Supplementary Information

Below is the link to the electronic supplementary material.

Supplementary Material 1

## Data Availability

All data generated or analysed during this study are included in this published article (and its Supplementary Information files).

## References

[1] Suskiewicz, M. J. The logic of protein post-translational modifications (PTMs): chemistry, mechanisms and evolution of protein regulation through covalent attachments. *BioEssays* **46** (3), 2300178. 10.1002/bies.202300178 (2024). 10.1002/bies.202300178 38247183

[2] Zhang, N. et al. Functions of lysine 2-hydroxyisobutyrylation and future perspectives on plants. *Proteomics* **23** (19), e2300045. 10.1002/pmic.202300045 (2023). 37338329 10.1002/pmic.202300045

[3] Ye, P. et al. Unlocking the brain’s code: the crucial role of post-translational modifications in neurodevelopment and neurological function. *Phys. Life Rev.* **53**, 187–214. 10.1016/j.plrev.2025.03.011 (2025). 40120399 10.1016/j.plrev.2025.03.011

[4] Lee, J. M. et al. Control of protein stability by post-translational modifications. *Nat. Commun.* **14** (1), 201. 10.1038/s41467-023-35795-8 (2023). 36639369 10.1038/s41467-023-35795-8 PMC9839724

[5] Peng, Y. et al. Targeted protein posttranslational modifications by chemically induced proximity for cancer therapy. *J. Biol. Chem.* **299** (4). 10.1016/j.jbc.2023.104572 (2023). 10.1016/j.jbc.2023.104572 PMC10050664 36870680

[6] Qin, Z. et al. Current computational tools for protein lysine acylation site prediction. *Brief. Bioinform.* **25** (6), bbae469. 10.1093/bib/bbae469 (2024). 39316944 10.1093/bib/bbae469 PMC11421846

[7] Dai, L. et al. Lysine 2-hydroxyisobutyrylation is a widely distributed active histone mark. *Nat. Chem. Biol.* **10** (5), 365–370. 10.1038/nchembio.1497 (2014). 24681537 10.1038/nchembio.1497

[8] Zhong, Q. et al. Protein posttranslational modifications in health and diseases: functions, regulatory mechanisms, and therapeutic implications. *MedComm* **2023** (4(3)), pe261. 10.1002/mco2.261 (2020). 10.1002/mco2.261 PMC10152985 37143582

[9] Yu, Z. et al. Proteome-wide identification of lysine 2-hydroxyisobutyrylation reveals conserved and novel histone modifications in physcomitrella patens. *Sci. Rep.* **7** (1), 15553. 10.1038/s41598-017-15854-z (2017). 29138512 10.1038/s41598-017-15854-z PMC5686104

[10] Larsen, M. R. et al. Analysis of posttranslational modifications of proteins by tandem mass spectrometry. *BioTechniques* **40** (6), 790–798. 10.2144/000112201 (2006). 16774123 10.2144/000112201

[11] Birhanu, A. G. Mass spectrometry-based proteomics as an emerging tool in clinical laboratories. *Clin. Proteomics*. **20** (1), 32. 10.1186/s12014-023-09424-x (2023). 37633929 10.1186/s12014-023-09424-x PMC10464495

[12] Meng, L. et al. Mini-review: recent advances in post-translational modification site prediction based on deep learning. *Comput. Struct. Biotechnol. J.* **20**, 3522–3532. 10.1016/j.csbj.2022.06.045 (2022). 35860402 10.1016/j.csbj.2022.06.045 PMC9284371

[13] Wang, D. et al. MusiteDeep: a deep-learning framework for general and kinase-specific phosphorylation site prediction. *Bioinformatics* **33** (24), 3909–3916. 10.1093/bioinformatics/btx496 (2017). 29036382 10.1093/bioinformatics/btx496 PMC5860086

[14] Luo, F. et al. DeepPhos: prediction of protein phosphorylation sites with deep learning. *Bioinformatics* **35** (16), 2766–2773. 10.1093/bioinformatics/bty1051 (2019). 30601936 10.1093/bioinformatics/bty1051 PMC6691328

[15] Fu, H. et al. DeepUbi: a deep learning framework for prediction of ubiquitination sites in proteins. *BMC Bioinform.* **20** (1), 86. 10.1186/s12859-019-2677-9 (2019). 10.1186/s12859-019-2677-9 PMC6379983 30777029

[16] Kirchoff, K. E. & Gomez, S. M. EMBER: multi-label prediction of kinase-substrate phosphorylation events through deep learning. *Bioinformatics* **38** (8), 2119–2126. 10.1093/bioinformatics/btac083 (2022). 35157015 10.1093/bioinformatics/btac083 PMC9004653

[17] Wang, H. et al. MDC-Kace: A model for predicting lysine acetylation sites based on modular densely connected convolutional networks. *IEEE Access.* **8**, 214469–214480. 10.1109/ACCESS.2020.3041044 (2020).

[18] Liu, Y. et al. DeepTL-Ubi: A novel deep transfer learning method for effectively predicting ubiquitination sites of multiple species. *Methods* **192**, 103–111. 10.1016/j.ymeth.2020.08.003 (2021). 32791338 10.1016/j.ymeth.2020.08.003

[19] Chandra, A. et al. Transformer-based deep learning for predicting protein properties in the life sciences. *eLife* **12**, e82819. 10.7554/eLife.82819 (2023). 36651724 10.7554/eLife.82819 PMC9848389

[20] Peng, F. Z. et al. PTM-Mamba: a PTM-aware protein Language model with bidirectional gated Mamba blocks. *Nat. Methods*. **22** (5), 945–949. 10.1038/s41592-025-02656-9 (2025). 40211004 10.1038/s41592-025-02656-9 PMC12074982

[21] Meng, L. et al. *UniPTM: Multiple PTM site prediction on full-length protein sequence.* bioRxiv, : p. 2024.08.03.606471 (2024). 10.1101/2024.08.03.606471

[22] Dipta, S. R. et al. SEMal: accurate protein malonylation site predictor using structural and evolutionary information. *Comput. Biol. Med.* **125**, 104022. 10.1016/j.compbiomed.2020.104022 (2020). 33022522 10.1016/j.compbiomed.2020.104022

[23] Arafat, M. E. et al. Accurate prediction of lysine methylation sites using evolutionary and Structural-Based information. *Cogn. Comput.* **16** (3), 1300–1320. 10.1007/s12559-024-10268-2 (2024).

[24] Wang, Y. G. et al. Accurate prediction of species-specific 2-hydroxyisobutyrylation sites based on machine learning frameworks. *Anal. Biochem.* **602**, 113793. 10.1016/j.ab.2020.113793 (2020). 32473122 10.1016/j.ab.2020.113793

[25] Ju, Z. & Wang, S. Y. iLys-Khib: identify lysine 2-Hydroxyisobutyrylation sites using mRMR feature selection and fuzzy SVM algorithm. *Chemometr. Intell. Lab. Syst.* **191**, 96–102. 10.1016/j.chemolab.2019.06.009 (2019).

[26] Zhang, L. et al. DeepKhib: A Deep-Learning framework for lysine 2-Hydroxyisobutyrylation sites prediction. *Front. Cell. Dev. Biology*. 10.3389/fcell.2020.580217 (2020). 10.3389/fcell.2020.580217 PMC7509169 33015075

[27] Jia, X. et al. ResNetKhib: a novel cell type-specific tool for predicting lysine 2-hydroxyisobutylation sites via transfer learning. *Brief. Bioinform.* **24** (2). 10.1093/bib/bbad063 (2023). 10.1093/bib/bbad063 PMC10185920 36880172

[28] Huang, H. et al. Landscape of the regulatory elements for lysine 2-hydroxyisobutyrylation pathway. *Cell Res.* **28** (1), 111–125. 10.1038/cr.2017.149 (2018). 29192674 10.1038/cr.2017.149 PMC5752845

[29] Wu, Q. et al. Global analysis of lysine 2-Hydroxyisobutyrylome upon SAHA treatment and its relationship with acetylation and crotonylation. *J. Proteome Res.* **17** (9), 3176–3183. 10.1021/acs.jproteome.8b00289 (2018). 30109935 10.1021/acs.jproteome.8b00289

[30] Lu, Y. et al. Global landscape of 2-hydroxyisobutyrylation in human pancreatic cancer. *Front. Oncol.* **12**, 1001807. 10.3389/fonc.2022.1001807 (2022). 36249039 10.3389/fonc.2022.1001807 PMC9563853

[31] Zhang, N. et al. *Global Profiling of 2-hydroxyisobutyrylome in Common Wheat.* Genomics, Proteomics & Bioinformatics, 20(4): pp. 688–701 (2022). 10.1016/j.gpb.2020.06.008 10.1016/j.gpb.2020.06.008 PMC9880814 33581340

[32] Bo, F. et al. Global analysis of lysine 2-hydroxyisobutyrylation in wheat root. *Sci. Rep.* **11** (1), 6327. 10.1038/s41598-021-85879-y (2021). 33737719 10.1038/s41598-021-85879-y PMC7973580

[33] Yin, D. et al. Global lysine crotonylation and 2-Hydroxyisobutyrylation in phenotypically different Toxoplasma gondii parasites. *Cell. Proteom.* **18** (11), 2207–2224. 10.1074/mcp.RA119.001611 (2019). Molecular. 10.1074/mcp.RA119.001611 PMC6823851 31488510

[34] Meng, X. et al. Proteome-wide analysis of lysine 2-hydroxyisobutyrylation in developing rice (Oryza sativa) seeds. *Sci. Rep.* **7** (1), 17486. 10.1038/s41598-017-17756-6 (2017). 29235492 10.1038/s41598-017-17756-6 PMC5727541

[35] Xue, C. et al. Proteome-Wide analyses reveal the diverse functions of lysine 2-Hydroxyisobutyrylation in Oryza sativa. *Rice* **13** (1), 34. 10.1186/s12284-020-00389-1 (2020). 32572646 10.1186/s12284-020-00389-1 PMC7310055

[36] Zheng, H. et al. Proteome-Wide analysis of lysine 2-Hydroxyisobutyrylation in Candida albicans. *mSystems* **6** (1). 10.1128/msystems.01129-20 (2021). 10.1128/mSystems.01129-20 PMC7857534 33531408

[37] Xu, Y. et al. Proteome-Wide analysis of lysine 2-Hydroxyisobutyrylation in the phytopathogenic fungus botrytis cinerea. *Front. Microbiol.* 11–2020. 10.3389/fmicb.2020.585614 (2020). 10.3389/fmicb.2020.585614 PMC7728723 33329453

[38] Fu, L. et al. CD-HIT: accelerated for clustering the next-generation sequencing data. *Bioinformatics* **28** (23), 3150–3152. 10.1093/bioinformatics/bts565 (2012). 23060610 10.1093/bioinformatics/bts565 PMC3516142

[39] Pourmirzaei, M. et al. Machine learning-based approaches for ubiquitination site prediction in human proteins. *BMC Bioinform.* **24** (1), 449. 10.1186/s12859-023-05581-w (2023). 10.1186/s12859-023-05581-w PMC10683244 38017391

[40] Tahir, M., Tayara, H. & Chong, K. T. iPseU-CNN: identifying RNA Pseudouridine sites using convolutional neural networks. *Mol. Therapy - Nucleic Acids*. **16**, 463–470. 10.1016/j.omtn.2019.03.010 (2019). 10.1016/j.omtn.2019.03.010 PMC6488737 31048185

[41] Ahmed, S. et al. DeepPPSite: A deep learning-based model for analysis and prediction of phosphorylation sites using efficient sequence information. *Anal. Biochem.* **612**, 113955. 10.1016/j.ab.2020.113955 (2021). 32949607 10.1016/j.ab.2020.113955

[42] Yu, B. et al. DNNAce: prediction of prokaryote lysine acetylation sites through deep neural networks with multi-information fusion. *Chemometr. Intell. Lab. Syst.* **200**, 103999. 10.1016/j.chemolab.2020.103999 (2020).

[43] Rives, A. et al. Biological structure and function emerge from scaling unsupervised learning to 250 million protein sequences. *Proc. Natl. Acad. Sci.* **118** (15), pe2016239118. 10.1073/pnas.2016239118 (2021). 10.1073/pnas.2016239118 PMC8053943 33876751

[44] Vieira, L. C., Handojo, M. L. & Wilke, C. O. *Medium-sized protein language models perform well at transfer learning on realistic datasets.* bioRxiv, : p. 2024.11.22.624936 (2025). 10.1101/2024.11.22.624936 10.1038/s41598-025-05674-x PMC12217344 40594749

[45] Rao, R. et al. MSA transformer. *BioRxiv* 20210212430858. 10.1101/2021.02.12.430858 (2021).

[46] Suzek, B. E. et al. UniRef clusters: a comprehensive and scalable alternative for improving sequence similarity searches. *Bioinformatics* **31** (6), 926–932. 10.1093/bioinformatics/btu739 (2015). 25398609 10.1093/bioinformatics/btu739 PMC4375400

[47] Lin, Z. et al. Evolutionary-scale prediction of atomic-level protein structure with a Language model. *Science* **379** (6637), 1123–1130. 10.1126/science.ade2574 (2023). 36927031 10.1126/science.ade2574

[48] Dubchak, I. et al. Prediction of protein folding class using global description of amino acid sequence. *Proc. Natl. Acad. Sci.* **92** (19), 8700–8704. 10.1073/pnas.92.19.8700 (1995). 7568000 10.1073/pnas.92.19.8700 PMC41034

[49] Kawashima, S. et al. AAindex: amino acid index database, progress report 2008. *Nucleic Acids Res.* **36** (suppl_1). 10.1093/nar/gkm998 (2008). p. D202-D205. 10.1093/nar/gkm998 PMC2238890 17998252

[50] Mohammadi, A. et al. PSSMCOOL: a comprehensive R package for generating evolutionary-based descriptors of protein sequences from PSSM profiles. *Biol. Methods Protoc.* **7** (1), bpac008. 10.1093/biomethods/bpac008 (2022). 35388370 10.1093/biomethods/bpac008 PMC8977839

[51] Altschul, S. F. et al. Gapped BLAST and PSI-BLAST: a new generation of protein database search programs. *Nucleic Acids Res.* **25** (17), 3389–3402. 10.1093/nar/25.17.3389 (1997). 9254694 10.1093/nar/25.17.3389 PMC146917

[52] Eddy, S. R. Where did the BLOSUM62 alignment score matrix come from? *Nat. Biotechnol.* **22** (8), 1035–1036. 10.1038/nbt0804-1035 (2004). 15286655 10.1038/nbt0804-1035

[53] Pietrokovski, S., Henikoff, J. G. & Henikoff, S. The blocks Database—A system for protein classification. *Nucleic Acids Res.* **24** (1), 197–200. 10.1093/nar/24.1.197 (1996). 8594578 10.1093/nar/24.1.197 PMC145590

[54] LeCun, Y. et al. Backpropagation applied to handwritten zip code recognition. *Neural Comput.* **1** (4), 541–551. 10.1162/neco.1989.1.4.541 (1989).

[55] Kiranyaz, S. et al. Convolutional neural networks for patient-specific ECG classification. *2015 37th Annual Int. Conf. IEEE Eng. Med. Biology Soc. (EMBC)*. 10.1109/EMBC.2015.7318926 (2015). 10.1109/EMBC.2015.7318926 26736826

[56] Vaz, J. M. & Balaji, S. Convolutional neural networks (CNNs): concepts and applications in pharmacogenomics. *Mol. Divers.* **25** (3), 1569–1584. 10.1007/s11030-021-10225-3 (2021). 34031788 10.1007/s11030-021-10225-3 PMC8342355

[57] Alzubaidi, L. et al. Review of deep learning: concepts, CNN architectures, challenges, applications, future directions. *J. Big Data*. **8** (1), 53. 10.1186/s40537-021-00444-8 (2021). 33816053 10.1186/s40537-021-00444-8 PMC8010506

[58] Yusuf, S. et al. DeepPPF: A deep learning framework for predicting protein family. *Neurocomputing* 428. 10.1016/j.neucom.2020.11.062 (2020).

[59] Cheng, J., Ying, X. & Zhao Y. and *Prediction of protein secondary structure based on deep residual convolutional neural network.* Biotechnology & Biotechnological Equipment, 35(1): pp. 1881–1890 (2021). 10.1080/13102818.2022.2026815

[60] Soleymani, F. et al. ProtInteract: A deep learning framework for predicting protein–protein interactions. *Comput. Struct. Biotechnol. J.* **21**, 1324–1348. 10.1016/j.csbj.2023.01.028 (2023). 36817951 10.1016/j.csbj.2023.01.028 PMC9929211

[61] Wen, B. et al. Deep learning in proteomics. *Proteomics* **20**, 21–22. 10.1002/pmic.201900335 (2020). 10.1002/pmic.201900335 PMC7757195 32939979

[62] Hahnloser, R. H. et al. Digital selection and analogue amplification coexist in a cortex-inspired silicon circuit. *Nature* **405** (6789), 947–951. 10.1038/35016072 (2000). 10879535 10.1038/35016072

[63] O’Malley, T. et al. *Keras tuner*. (2019).

[64] Kingma, D. P. & Ba, J. *Adam: A method for stochastic optimization*. *ArXiv Preprint arXiv* :1412.6980, 2014 10.48550/arXiv.1412.6980

[65] Pettit, R. W. et al. Artificial intelligence, machine learning, and deep learning for clinical outcome prediction. *Emerg. Top. Life Sci.* **5** (6), 729–745. 10.1042/ETLS20210246 (2021). 34927670 10.1042/ETLS20210246 PMC8786279

[66] Lipton, Z. *A Critical Review of Recurrent Neural Networks for Sequence Learning.* (2015). 10.48550/arXiv.1506.00019

[67] Hochreiter, S. & Schmidhuber, J. Long short-term memory. *Neural Comput.* **9** (8), 1735–1780. 10.1162/neco.1997.9.8.1735 (1997). 9377276 10.1162/neco.1997.9.8.1735

[68] Cho, K. et al. *Learning Phrase Representations using RNN Encoder-Decoder for Statistical Machine Translation.* (2014). 10.48550/arXiv.1406.1078

[69] Schuster, M. & Paliwal, K. K. Bidirectional recurrent neural networks. *IEEE Trans. Signal Process.* **45** (11), 2673–2681 (1997).

[70] Wong, T. T. Performance evaluation of classification algorithms by k-fold and leave-one-out cross validation. *Pattern Recogn.* **48** (9), 2839–2846. 10.1016/j.patcog.2015.03.009 (2015).

[71] Trucco, E. et al. in *Chap. 9 - Validation. Computational Retinal Image Analysis*. 157–170 (eds Trucco, E., MacGillivray, T. & Xu, Y.) (Academic, 2019). 10.1016/B978-0-08-102816-2.00009-5

[72] Vacic, V., Iakoucheva, L. M. & Radivojac, P. Two sample logo: a graphical representation of the differences between two sets of sequence alignments. *Bioinformatics* **22** (12), 1536–1537. 10.1093/bioinformatics/btl151 (2006). 16632492 10.1093/bioinformatics/btl151

[73] Xie, J. et al. DeepMPSF: A deep learning network for predicting general protein phosphorylation sites based on multiple protein sequence features. *J. Chem. Inf. Model.* **63** (22), 7258–7271. 10.1021/acs.jcim.3c00996 (2023). 37931253 10.1021/acs.jcim.3c00996

[74] Taunk, K. et al. *A Brief Review of Nearest Neighbor Algorithm for Learning and Classification*. in. *International Conference on Intelligent Computing and Control Systems (ICCS)*. 2019. (2019).

[75] Cortes, C. & Vapnik, V. Support-vector networks. *Mach. Learn.* **20** (3), 273–297. 10.1007/BF00994018 (1995).

[76] Breiman, L. Random forests. *Mach. Learn.* **45** (1), 5–32. 10.1023/A:1010933404324 (2001).

[77] Chen, T. & Guestrin, C. *XGBoost: A Scalable Tree Boosting System*. 785–794 (2016). 10.1145/2939672.2939785

[78] Ke, G. et al. *LightGBM: a highly efficient gradient boosting decision tree*. in *Proceedings of the 31st International Conference on Neural Information Processing Systems*. Long Beach, California, USA: Curran Associates Inc. (2017). https://api.semanticscholar.org/CorpusID:3815895

[79] Prokhorenkova, L. et al. *CatBoost: unbiased boosting with categorical features*. in *Proceedings of the 32nd International Conference on Neural Information Processing Systems*. Montréal, Canada: Curran Associates Inc. (2018). https://arxiv.org/abs/1706.09516

