# Supplementary material for: A deep learning framework for lysine 2-hydroxyisobutyrylation site prediction using evolutionary feature representation
Source: Sci Rep. 2025 Nov 6;15:38838. doi: 10.1038/s41598-025-15883-z (PMC12592433; doi:10.1038/s41598-025-15883-z)
Supplement: Supplementary file 1 — Supplementary Material 1 [file 41598_2025_15883_MOESM1_ESM.docx]

| **Table S1.** List of amino acid indices utilized in the study, along with their corresponding descriptions**.** | |
| --- | --- |
| **AAindex** | **Description** |
| EISD860102 | Atom-based hydrophobic moment |
| BIGC670101 | Residue volume |
| CHAM820101 | Polarizability parameter |
| CHAM830101 | The Chou-Fasman parameter of the coil conformation |
| PALJ810101 | Normalized frequency of alpha-helix from LG |
| KYTJ820101 | Hydropathy index |
| EISD860101 | Solvation free energy |
| ZIMJ680102 | Bulkiness |
| VINM940101 | Normalized flexibility parameters (B-values) |
| ZIMJ680104 | Isoelectric point |
| BHAR880101 | Average flexibility indices |
| LEVM780101 | Normalized frequency of alpha-helix, with weights |
| CHAM830107 | A parameter of charge transfer capability |
| KLEP840101 | Net charge |
| KRIW790103 | Side chain volume |
| FAUJ830101 | Hydrophobic parameter pi |
| KRIW790102 | Fraction of site occupied by water |
| DAYM780101 | Amino acid composition |
| ROSM880102 | Side chain hydropathy, corrected for solvation |
| JUKT750101 | Amino acid distribution |
| PRAM900102 | Relative frequency in alpha-helix |
| TAKK010101 | Side-chain contribution to protein stability (kJ/mol) |

| **Table S2.** Optimized CNN model architectures across various window sizes for Khib site prediction. | | | | | | | |
| --- | --- | --- | --- | --- | --- | --- | --- |
| **Window**  **size**  **Model**  **architecture** | **47** | **45** | **43** | **41** | **39** | **37** | **35** |
| **Number of convolution layers** | 2 | 6 | 6 | 3 | 2 | 3 | 1 |
| **(Number of filters, Kernel size)** | (256,7)  (32,5) | (512,5)  (160,3)  (384,3)  (64,5)  (288,9)  (288,9) | (265,7)  (384,7)  (320,7)  (288,7)  (512,3)  (128,5) | (480,5)  (128,7)  (128,5) | (128,3)  (224,5) | (352,7)  (96,7)  (32,3) | (320,7) |
| **Activation function** | ReLU | ReLU | ReLU | ReLU | ReLU | ReLU | ReLU |
| **Max pooling (size=2)** | 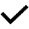 | 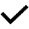 | 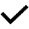 | 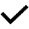 | 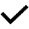 | 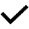 | 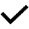 |
| **Dropout/Convolution layer** | 0.4  0.3 | 0.4  0.1  0.3  0.0  0.3  0.4 | 0.0  0.0  0.0  0.1  0.1  0.0 | 0.4  0.0  0.4 | 0.3  0.4 | 0.4  0.0  0.0 | 0.2 |
| **Number of dense layers** | 4 | 4 | 1 | 3 | 1 | 3 | 3 |
| **Number of units/ dense layer** | 256  512  352  96 | 384  480  352  480 | 384 | 512  192  64 | 256 | 160  128  448 | 384  96  64 |
| **Dropout/ dense layer** | 0.2  0.2  0.2  0.1 | 0.1  0.3  0.2  0.0 | 0.0 | 0.1  0.1  0.4 | 0.1 | 0.2  0.3  0.3 | 0.2  0.2  0.2 |
| **Learning rate** | 0.0001 | 0.001 | 0.0001 | 0.001 | 0.0001 | 0.0001 | 0.0001 |

| **Table S3.** Optimal CNN hyperparameter configurations for different feature representation methods for Khib site prediction. | | | | | | |
| --- | --- | --- | --- | --- | --- | --- |
| **Feature**  **representation**  **method**  **Model**  **architecture** | **ESM** | **One-hot** | **CTD** | **PSSM** | **AAP** | **BLOSUM** |
| **Number of convolution layers** | 1 | 1 | 2 | 4 | 3 | 6 |
| **(Number of filters, Kernel size)** | (256,7) | (224,7) | (320,5)  (480,7) | (32,3)  (512,3)  (160,5)  (224,3) | (480,9)  (192,5)  (416,5) | (256,7)  (384,7)  (320,7)  (288,7)  (512,3)  (128,5) |
| **Activation function** | ReLU | ReLU | ReLU | ReLU | ReLU | ReLU |
| **Max pooling (size=2)** | 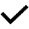 | 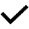 | 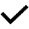 | 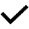 | 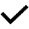 | 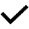 |
| **Dropout / Convolution layer** | 0.4 | 0.4 | 0.2  0.3 | 0.1  0.1  0.3  0.4 | 0.1  0.4  0.1 | 0.0  0.0  0.0  0.1  0.1  0.0 |
| **Number of dense layers** | 1 | 4 | 4 | 3 | 1 | 1 |
| **Number of units / dense layer** | 96 | 288  32  32  32 | 320  32  32  32 | 256  448  192 | 192 | 384 |
| **Dropout / dense layer** | 0.1 | 0.2  0.0  0.0  0.0 | 0.1  0.0  0.0  0.0 | 0.3  0.1  0.0 | 0.1 | 0.0 |
| **Learning rate** | 0.001 | 0.0001 | 0.001 | 0.001 | 0.0001 | 0.0001 |

| 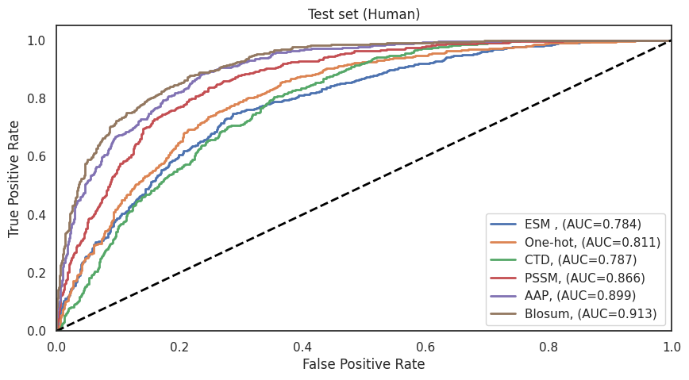 | 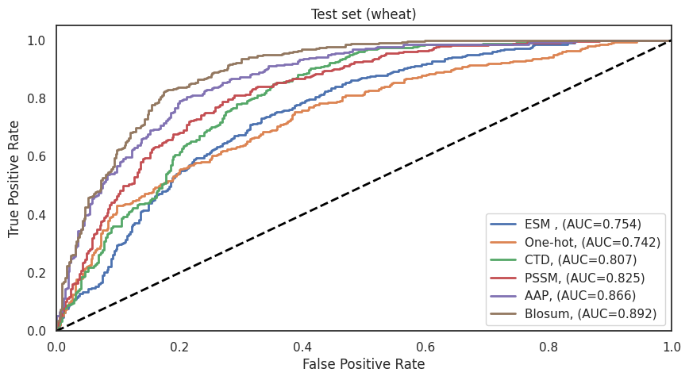 |
| --- | --- |
| (a) | (b) |
| 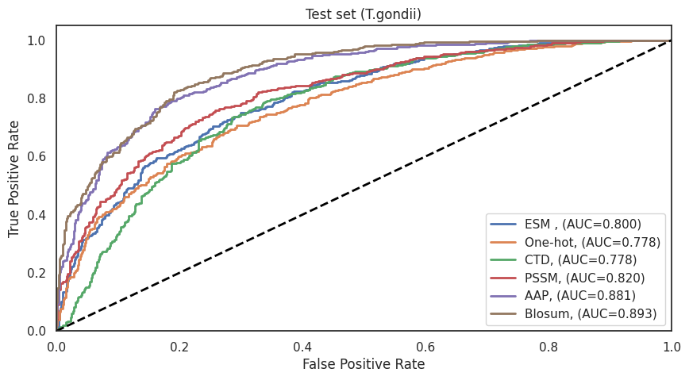 | 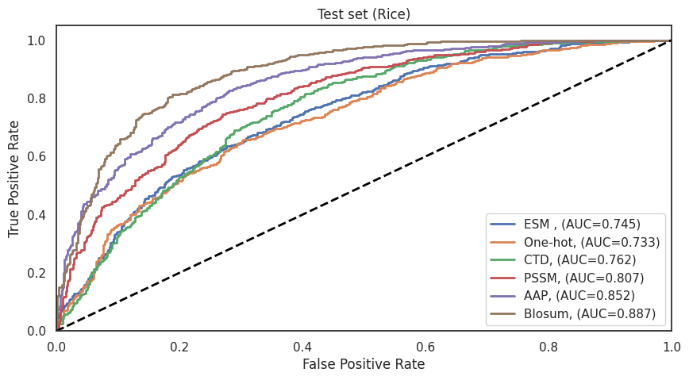 |
| (c) | (d) |
| 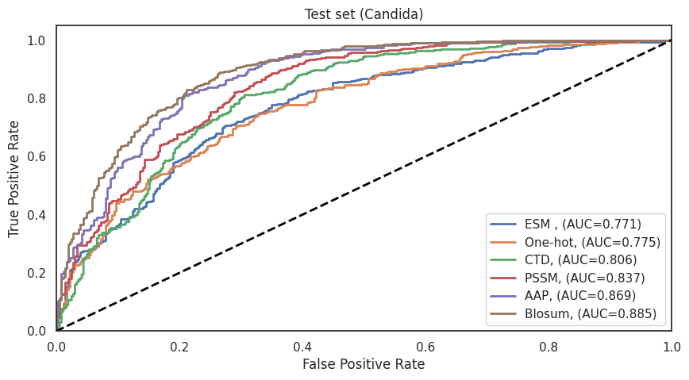 | 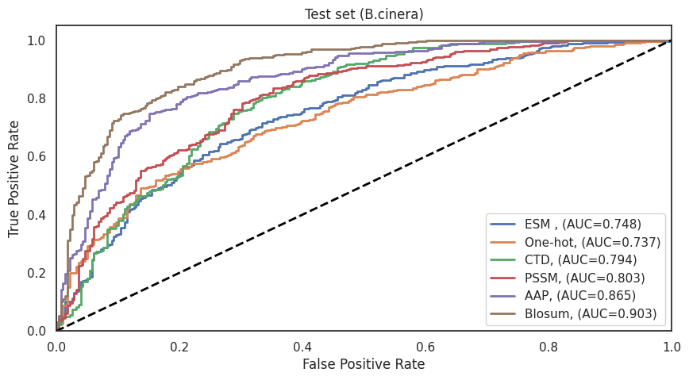 |
| (e) | (f) |
| **Fig. S1** ROC curves for different feature representation methods in Khib site prediction using their respective optimized CNN architectures on the independent test sets of the (a) human, (b) wheat, (c) *T. gondii*, (d) rice, (e) Candida, and (f) *B. cinerea* datasets. | |

| **Table S4.** Optimal CNN hyperparameter configurations for different feature fusion methods for Khib site prediction. | | | | | |
| --- | --- | --- | --- | --- | --- |
| **Fused**  **features**  **Model**  **architecture** | **BLOSUM+**  **ESM** | **BLOSUM+**  **onehot** | **BLOSUM+**  **CTD** | **BLOSUM+**  **PSSM** | **BLOSUM+**  **AAP** |
| **Number of convolution layers** | 2 | 4 | 3 | 4 | 6 |
| **Number of filters/ layers,**  **Kernel size)** | (512,5)  (128,5) | (352,7)  (160,7)  (352,9)  (64,5) | (32,9)  (352,7)  (352,3) | (480,9)  (416,3)  (224,5)  (224,9) | (480,9)  (480,5)  (160,9)  (128,3)  (32,3)  (192,7) |
| **Activation function** | ReLU | ReLU | ReLU | ReLU | ReLU |
| **Max pooling**  **/Convolution layer** | 3  4 | 2  2  2  2 | 4  4  2 | 3  3  2  3 | 3  3  3  3  3  3 |
| **Dropout**  **/Convolution layer** | 0.0  0.2 | 0.0  0.3  0.2  0.2 | 0.4  0.0  0.0 | 0.2  0.2  0.4  0.1 | 0.0  0.1  0.0  0.2  0.1  0.3 |
| **Number of dense layers** | 4 | 4 | 3 | 1 | 1 |
| **Number of units/ dense layer** | 64  480  416  32 | 64  32  96  512 | 416  384  224 | 256 | 160 |
| **Dropout/ dense layer** | 0.0  0.4  0.3  0.3 | 0.0  0.3  0.0  0.2 | 0.3  0.0  0.1 | 0.1 | 0.0 |
| **Learning rate** | 0.0001 | 0.0001 | 0.001 | 0.001 | 0.0001 |

| **Table S5.** Comparative performance of feature fusion methods for Khib site prediction in the human dataset using their respective optimized CNN architectures. | | | | | | | | | |
| --- | --- | --- | --- | --- | --- | --- | --- | --- | --- |
| **Feature fusion method** | **10-fold cross-validation set** | | | |  | **Independent test set** | | | |
|  | **ACC** | **F1** | **MCC** | **AUC** |  | **ACC** | **F1** | **MCC** | **AUC** |
| **BLSOUM+ESM** | 0.808 | 0.814 | 0.618 | 0.886 |  | 0.814 | 0.816 | 0.627 | 0.897 |
| **BLOSUM+one hot** | 0.808 | 0.814 | 0.617 | 0.880 |  | 0.803 | 0.816 | 0.610 | 0.888 |
| **BLOSUM+CTD** | 0.812 | 0.821 | 0.628 | 0.881 |  | 0.801 | 0.812 | 0.604 | 0.895 |
| **BLSOUM+PSSM** | 0.800 | 0.807 | 0.602 | 0.881 |  | 0.809 | 0.814 | 0.618 | 0.891 |
| **BLOSUM+AAP** | 0.811 | 0.819 | 0.624 | 0.884 |  | 0.799 | 0.805 | 0.598 | 0.885 |

| **Table S6.** Comparative performance of feature fusion methods for Khib site prediction in the wheat dataset using their respective optimized CNN architectures. | | | | | | | | | |
| --- | --- | --- | --- | --- | --- | --- | --- | --- | --- |
| **Feature fusion method** | **10-fold cross-validation set** | | | |  | **Independent test set** | | | |
|  | **ACC** | **F1** | **MCC** | **AUC** |  | **ACC** | **F1** | **MCC** | **AUC** |
| **BLSOUM+ESM** | 0.791 | 0.794 | 0.582 | 0.859 |  | 0.772 | 0.772 | 0.543 | 0.862 |
| **BLOSUM+one hot** | 0.756 | 0.784 | 0.530 | 0.846 |  | 0.773 | 0.787 | 0.548 | 0.865 |
| **BLOSUM+CTD** | 0.779 | 0.792 | 0.563 | 0.853 |  | 0.781 | 0.797 | 0.566 | 0.859 |
| **BLSOUM+PSSM** | 0.807 | 0.811 | 0.614 | 0.864 |  | 0.783 | 0.792 | 0.567 | 0.855 |
| **BLOSUM+AAP** | 0.769 | 0.772 | 0.538 | 0.853 |  | 0.760 | 0.766 | 0.520 | 0.845 |

| **Table S7.** Comparative performance of feature fusion methods for Khib site prediction in the *T. gondii* dataset using their respective optimized CNN architectures. | | | | | | | | | |
| --- | --- | --- | --- | --- | --- | --- | --- | --- | --- |
| **Feature fusion method** | **10-fold cross-validation set** | | | |  | **Independent test set** | | | |
|  | **ACC** | **F1** | **MCC** | **AUC** |  | **ACC** | **F1** | **MCC** | **AUC** |
| **BLSOUM+ESM** | 0.792 | 0.797 | 0.586 | 0.877 |  | 0.790 | 0.783 | 0.580 | 0.878 |
| **BLOSUM+one hot** | 0.778 | 0.781 | 0.557 | 0.864 |  | 0.782 | 0.779 | 0.563 | 0.872 |
| **BLOSUM+CTD** | 0.792 | 0.794 | 0.585 | 0.876 |  | 0.793 | 0.795 | 0.588 | 0.881 |
| **BLSOUM+PSSM** | 0.787 | 0.790 | 0.574 | 0.873 |  | 0.782 | 0.778 | 0.565 | 0.876 |
| **BLOSUM+AAP** | 0.757 | 0.750 | 0.511 | 0.847 |  | 0.771 | 0.769 | 0.543 | 0.858 |

| **Table S8.** Comparative performance of feature fusion methods for Khib site prediction in the rice dataset using their respective optimized CNN architectures. | | | | | | | | | |  |
| --- | --- | --- | --- | --- | --- | --- | --- | --- | --- | --- |
| **Feature fusion method** | **10-fold cross-validation set** | | | |  | **Independent test set** | | | | |
|  | **ACC** | **F1** | **MCC** | **AUC** |  | **ACC** | **F1** | **MCC** | **AUC** | |
| **BLSOUM+ESM** | 0.772 | 0.778 | 0.546 | 0.851 |  | 0.756 | 0.764 | 0.512 | 0.849 | |
| **BLOSUM+one hot** | 0.762 | 0.769 | 0.525 | 0.839 |  | 0.744 | 0.751 | 0.488 | 0.839 | |
| **BLOSUM+CTD** | 0.771 | 0.780 | 0.545 | 0.849 |  | 0.770 | 0.778 | 0.540 | 0.853 | |
| **BLSOUM+PSSM** | 0.769 | 0.779 | 0.541 | 0.848 |  | 0.777 | 0.799 | 0.557 | 0.856 | |
| **BLOSUM+AAP** | 0.763 | 0.772 | 0.529 | 0.843 |  | 0.757 | 0.768 | 0.513 | 0.845 | |

| **Table S9.** Comparative performance of feature fusion methods for Khib site prediction in the Candida dataset using their respective optimized CNN architectures. | | | | | | | | | |
| --- | --- | --- | --- | --- | --- | --- | --- | --- | --- |
| **Feature fusion method** | **10-fold cross-validation set** | | | |  | **Independent test set** | | | |
|  | **ACC** | **F1** | **MCC** | **AUC** |  | **ACC** | **F1** | **MCC** | **AUC** |
| **BLSOUM+ESM** | 0.790 | 0.799 | 0.584 | 0.874 |  | 0.792 | 0.796 | 0.583 | 0.869 |
| **BLOSUM+one hot** | 0.776 | 0.791 | 0.559 | 0.857 |  | 0.785 | 0.788 | 0.570 | 0.873 |
| **BLOSUM+CTD** | 0.782 | 0.794 | 0.569 | 0.866 |  | 0.789 | 0.808 | 0.585 | 0.873 |
| **BLSOUM+PSSM** | 0.781 | 0.791 | 0.565 | 0.860 |  | 0.785 | 0.793 | 0.570 | 0.870 |
| **BLOSUM+AAP** | 0.778 | 0.792 | 0.563 | 0.858 |  | 0.763 | 0.779 | 0.528 | 0.855 |

| **Table S10.** Comparative performance of feature fusion methods for Khib site prediction in the *B. cinerea* dataset using their respective optimized CNN architectures. | | | | | | | | | |
| --- | --- | --- | --- | --- | --- | --- | --- | --- | --- |
| **Feature fusion method** | **10-fold cross-validation set** | | | |  | **Independent test set** | | | |
|  | **ACC** | **F1** | **MCC** | **AUC** |  | **ACC** | **F1** | **MCC** | **AUC** |
| **BLSOUM+ESM** | 0.785 | 0.792 | 0.573 | 0.862 |  | 0.809 | 0.822 | 0.617 | 0.873 |
| **BLOSUM+one hot** | 0.777 | 0.787 | 0.559 | 0.853 |  | 0.795 | 0.816 | 0.584 | 0.862 |
| **BLOSUM+CTD** | 0.786 | 0793 | 0.575 | 0.866 |  | 0.788 | 0.802 | 0.576 | 0.864 |
| **BLSOUM+PSSM** | 0.783 | 0.789 | 0.569 | 0.853 |  | 0.786 | 0.795 | 0.577 | 0.865 |
| **BLOSUM+AAP** | 0.766 | 0.774 | 0.536 | 0.840 |  | 0.748 | 0.766 | 0.493 | 0.828 |

| 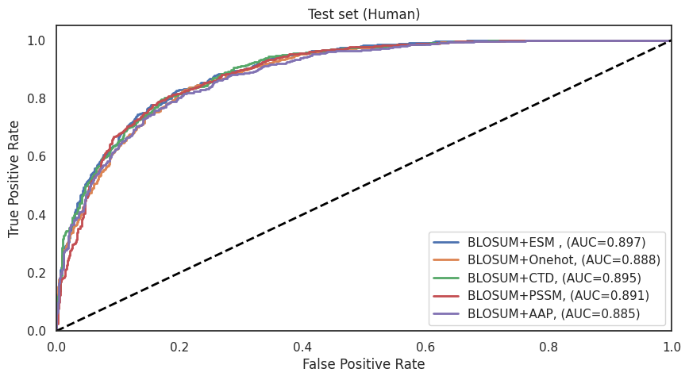 | 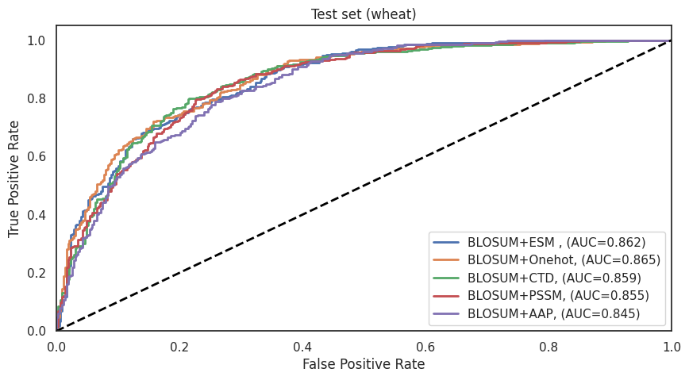 |
| --- | --- |
| (a) | (b) |
| 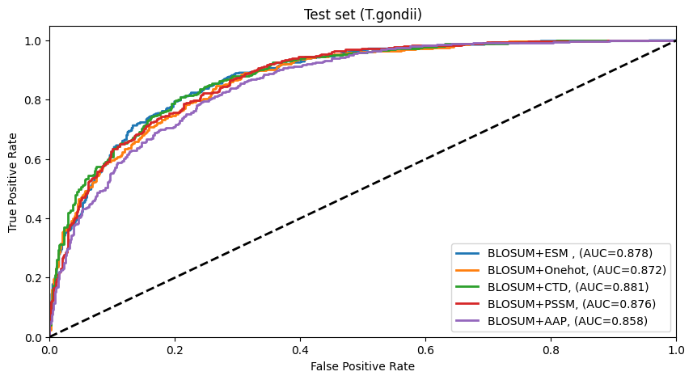 | 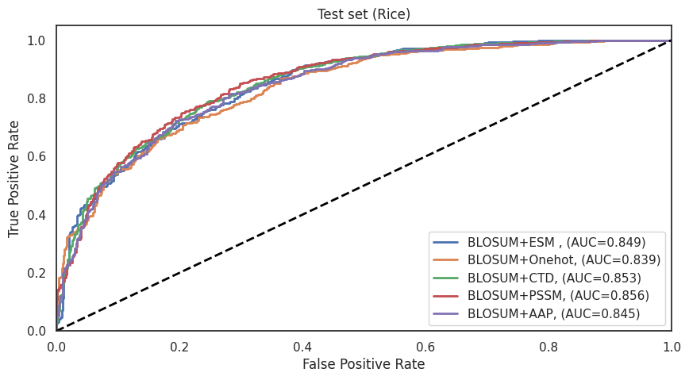 |
| (c) | (d) |
| 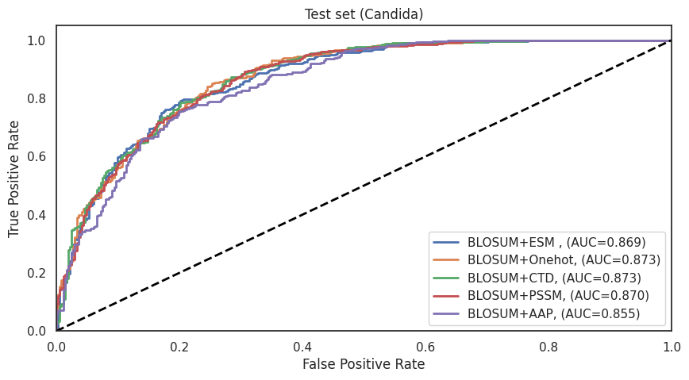 | 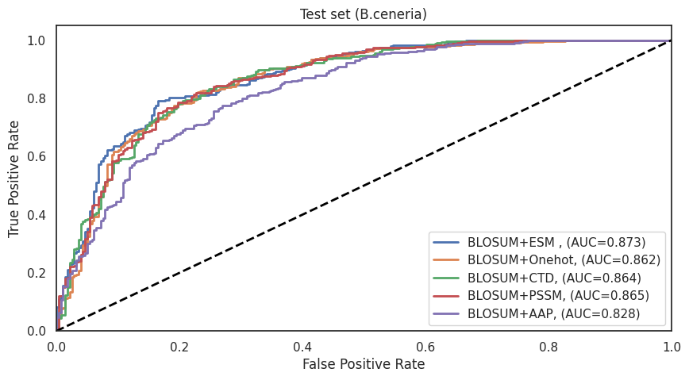 |
| (e) | (f) |
| **Fig. S2** ROC curves demonstrating the discriminative performance of various feature fusion strategies Khib site prediction using their respective optimized CNN architectures across the independent test sets of the (a) human, (b) wheat, (c) *T.* *gondii*, (d) rice, (e) Candida, and (f) *B. cinerea* datasets. | |

| **Table S11.** Optimized architectures of deep learning models used for Khib site prediction. | | | | | |
| --- | --- | --- | --- | --- | --- |
| **Model**  **type**  **Architecture** | **DNN** | **LSTM** | **GRU** | **Bi-LSTM** | **Bi-GRU** |
| **Number of layers** | 1 | 3 | 2 | 2 | 1 |
| **Number of neurons/layers** | 256 | 256  32  416 | 128  64 | 416  192 | 96 |
| **Batch normalization** | 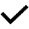 | 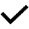 | 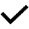 | 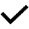 | 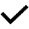 |
| **Activation function** | ReLU | ReLU | ReLU | ReLU | ReLU |
| **Global max pooling 1d** | - | - | - | 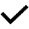 | 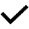 |
| **Dropout/layer** | 0.3 | 0.2  0.4  0.2 | 0.3  0.1 | 0.3  0.1 | 0.5 |
| **Learning rate** | 0.001 | 0.001 | 0.001 | 0.001 | 0.001 |
| **Number of epochs** | 60 | 100 | 90 | 60 | 60 |

| **Table S12**. Performance comparison of different deep learning models for Khib site prediction on the human dataset. | | | | | | | | | |
| --- | --- | --- | --- | --- | --- | --- | --- | --- | --- |
| **Model** | **10-fold cross-validation** | | | |  | **Independent test set** | | | |
|  | **ACC** | **F1** | **MCC** | **AUC** |  | **ACC** | **F1** | **MCC** | **AUC** |
| **DNN** | 0.738 | 0.742 | 0.477 | 0.811 |  | 0.745 | 0.755 | 0.490 | 0.827 |
| **GRU** | 0.819 | 0.825 | 0.640 | 0.899 |  | 0.814 | 0.815 | 0.627 | 0.901 |
| **BiGRU** | 0.815 | 0.824 | 0.635 | 0.896 |  | 0.830 | 0.836 | 0.660 | 0.904 |
| **LSTM** | 0.798 | 0.807 | 0.599 | 0.873 |  | 0.812 | 0.822 | 0.626 | 0.892 |
| **BiLSTM** | 0.817 | 0.822 | 0.635 | 0.893 |  | 0.808 | 0.814 | 0.617 | 0.896 |
| **BLOS-Khib** | **0.818** | **0.825** | **0.640** | **0.902** |  | **0.823** | **0.837** | **0.653** | **0.913** |

| **Table S13.** Performance comparison of different deep learning models for Khib site prediction on the wheat dataset. | | | | | | | | | |
| --- | --- | --- | --- | --- | --- | --- | --- | --- | --- |
| **Model** | **10-fold cross-validation** | | | | **Independent test set** | | | | |
|  | **ACC** | **F1** | **MCC** | **AUC** |  | **ACC** | **F1** | **MCC** | **AUC** |
| **DNN** | 0.687 | 0.687 | 0.377 | 0.750 |  | 0.678 | 0.668 | 0.357 | 0.732 |
| **GRU** | 0.812 | 0.819 | 0.630 | 0.890 |  | 0.798 | 0.801 | 0.596 | 0.882 |
| **BiGRU** | 0.801 | 0.807 | 0.605 | 0.885 |  | 0.799 | 0.798 | 0.599 | 0.886 |
| **LSTM** | 0.718 | 0.702 | 0.440 | 0.797 |  | 0.784 | 0.796 | 0.570 | 0.870 |
| **BiLSTM** | 0.807 | 0.815 | 0.617 | 0.888 |  | 0.805 | 0.822 | 0.619 | 0.881 |
| **BLOS-Khib** | **0.810** | **0.817** | **0.626** | **0.890** |  | **0.790** | **0.777** | **0.586** | **0.892** |

| **Table S14.** Performance comparison of different deep learning models for Khib site prediction on the *T. gondii* dataset. | | | | | | | | | |
| --- | --- | --- | --- | --- | --- | --- | --- | --- | --- |
| **Model** | **10-fold cross-validation** | | | |  | **Independent test set** | | | |
|  | **ACC** | **F1** | **MCC** | **AUC** |  | **ACC** | **F1** | **MCC** | **AUC** |
| **DNN** | 0.713 | 0.722 | 0.429 | 0.792 |  | 0.727 | 0.728 | 0.454 | 0.806 |
| **GRU** | 0.813 | 0.820 | 0.629 | 0.894 |  | 0.787 | 0.777 | 0.573 | 0.881 |
| **BiGRU** | 0.804 | 0.820 | 0.608 | 0.883 |  | 0.787 | 0.777 | 0.573 | 0.881 |
| **LSTM** | 0.777 | 0.784 | 0.558 | 0.862 |  | 0.782 | 0.781 | 0.565 | 0.868 |
| **BiLSTM** | 0.813 | 0.817 | 0.630 | 0.893 |  | 0.796 | 0.801 | 0.595 | 0.886 |
| **BLOS-Khib** | **0.815** | **0.822** | **0.634** | **0.896** |  | **0.804** | **0.800** | **0.609** | **0.893** |

| **Table S15.** Performance comparison of different deep learning models for Khib site prediction on the rice dataset. | | | | | | | | | |
| --- | --- | --- | --- | --- | --- | --- | --- | --- | --- |
| **Model** | **10-fold cross-validation** | | | |  | **Independent test set** | | | |
|  | **ACC** | **F1** | **MCC** | **AUC** |  | **ACC** | **F1** | **MCC** | **AUC** |
| **DNN** | 0.685 | 0.681 | 0.373 | 0.752 |  | 0.683 | 0.717 | 0.367 | 0.748 |
| **GRU** | 0.776 | 0.782 | 0.554 | 0.853 |  | 0.790 | 0.794 | 0.581 | 0.871 |
| **BiGRU** | 0.781 | 0.784 | 0.566 | 0.865 |  | 0.777 | 0.801 | 0.558 | 0.875 |
| **LSTM** | 0.748 | 0.732 | 0.503 | 0.835 |  | 0.751 | 0.745 | 0.509 | 0.837 |
| **BiLSTM** | 0.780 | 0.785 | 0.564 | 0.863 |  | 0.789 | 0.799 | 0.577 | 0.872 |
| **BLOS-Khib** | **0.785** | **0.794** | **0.577** | **0.869** |  | **0.807** | **0.822** | **0.614** | **0.887** |

| **Table S16.** Performance comparison of different deep learning models for Khib site prediction on the Candida dataset. | | | | | | | | | |
| --- | --- | --- | --- | --- | --- | --- | --- | --- | --- |
| **Model** | **10-fold cross-validation** | | | |  | **Independent test set** | | | |
|  | **ACC** | **F1** | **MCC** | **AUC** |  | **ACC** | **F1** | **MCC** | **AUC** |
| **DNN** | 0.701 | 0.698 | 0.404 | 0.778 |  | 0.698 | 0.689 | 0.399 | 0.785 |
| **GRU** | 0.785 | 0.791 | 0.575 | 0.865 |  | 0.773 | 0.769 | 0.547 | 0.857 |
| **BiGRU** | 0.801 | 0.809 | 0.605 | 0.877 |  | 0.784 | 0.792 | 0.567 | 0.860 |
| **LSTM** | 0.728 | 0.757 | 0.473 | 0.806 |  | 0.769 | 0.795 | 0.549 | 0.835 |
| **BiLSTM** | 0.788 | 0.797 | 0.581 | 0.872 |  | 0.769 | 0.774 | 0.537 | 0.872 |
| **BLOS-Khib** | **0.804** | **0.809** | **0.611** | **0.886** |  | **0.801** | **0.803** | **0.602** | **0.885** |

| **Table S17.** Performance comparison of different deep learning models for Khib site prediction on the *B. cinerea* dataset. | | | | | | | | | |
| --- | --- | --- | --- | --- | --- | --- | --- | --- | --- |
| **Model** | **10-fold cross-validation** | | | |  | **Independent test set** | | | |
|  | **ACC** | **F1** | **MCC** | **AUC** |  | **ACC** | **F1** | **MCC** | **AUC** |
| **DNN** | 0.686 | 0.672 | 0.373 | 0.743 |  | 0.693 | 0.721 | 0.379 | 0.739 |
| **GRU** | 0.791 | 0.798 | 0.588 | 0.870 |  | 0.775 | 0.786 | 0.553 | 0.868 |
| **BiGRU** | 0.799 | 0.803 | 0.602 | 0.876 |  | 0.814 | 0.836 | 0.623 | 0.892 |
| **LSTM** | 0.748 | 0.761 | 0.505 | 0.820 |  | 0.774 | 0.802 | 0.540 | 0.841 |
| **BiLSTM** | 0.784 | 0.794 | 0.578 | 0.869 |  | 0.793 | 0.798 | 0.594 | 0.891 |
| **BLOS-Khib** | **0.800** | **0.806** | **0.604** | **0.882** |  | **0.819** | **0.833** | **0.635** | **0.903** |

| 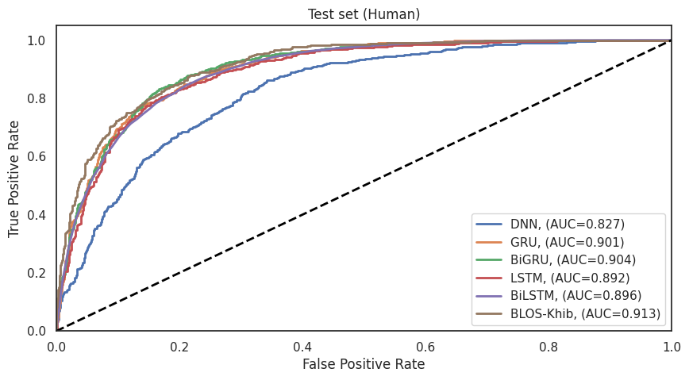 | 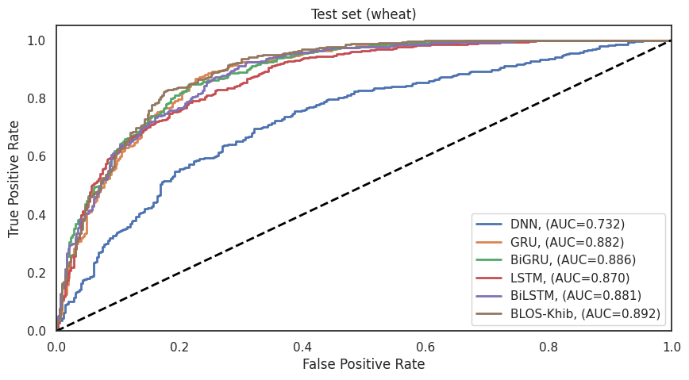 |
| --- | --- |
| (a) | (b) |
| 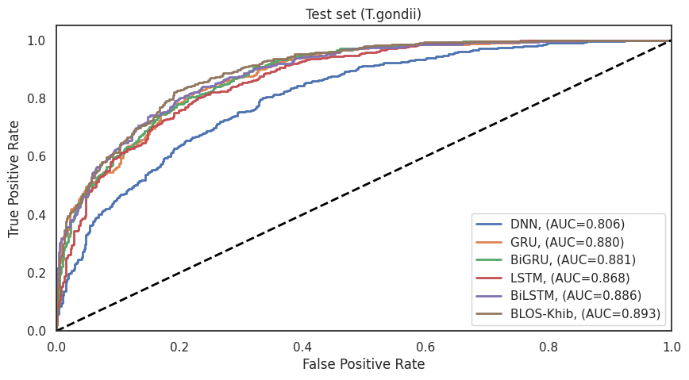 | 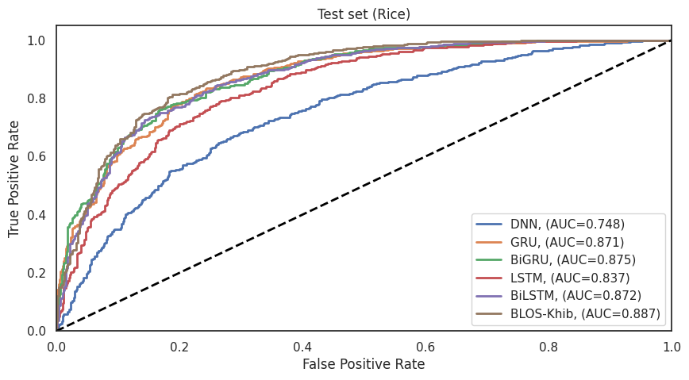 |
| (c) | (d) |
| 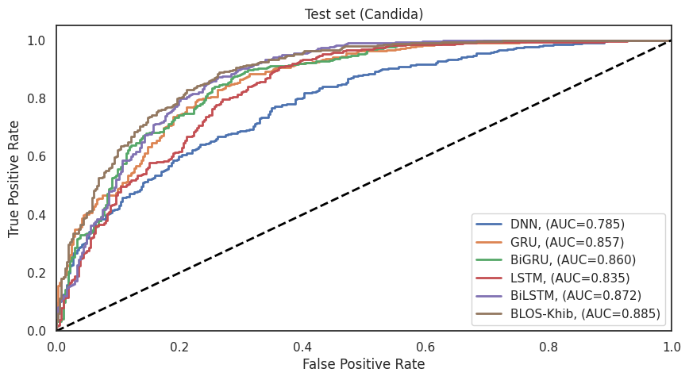 | 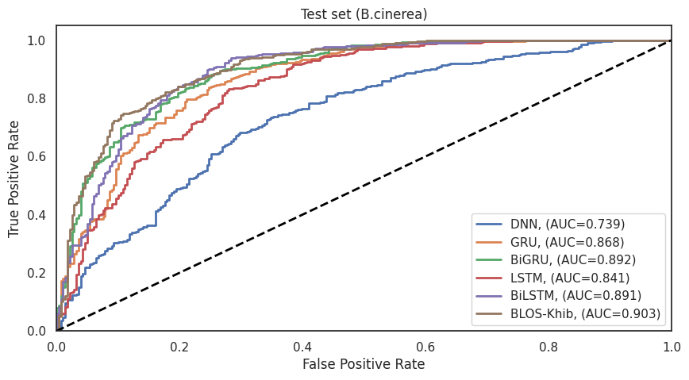 |
| (e) | (f) |
| **Fig. S3** ROC curves comparing optimized deep learning models with BLOS-Khib on the independent test sets from six datasets: (a) human, (b) wheat, (c) *T. gondii*, (d) rice, (e) Candida, and (f) *B. cinerea*. | |

| **Table S18.** Performance comparison of machine learning classifiers with BLOS-Khib for Khib site prediction on the human dataset. | | | | | | | | | |
| --- | --- | --- | --- | --- | --- | --- | --- | --- | --- |
| **Classifier** | **10-fold cross-validation** | | | |  | **Independent test set** | | | |
|  | **ACC** | **F1** | **MCC** | **AUC** |  | **ACC** | **F1** | **MCC** | **AUC** |
| **KNN** | 0.585 | 0.622 | 0.173 | 0.625 |  | 0.594 | 0.632 | 0.190 | 0.630 |
| **SVM** | 0.719 | 0.718 | 0.439 | 0.791 |  | 0.730 | 0.732 | 0.461 | 0.805 |
| **RF** | 0.740 | 0.743 | 0.479 | 0.819 |  | 0.747 | 0.753 | 0.494 | 0.825 |
| **XGBoost** | 0.778 | 0.781 | 0.557 | 0.857 |  | 0.780 | 0.787 | 0.561 | 0.865 |
| **LightGBM** | 0.780 | 0.783 | 0.561 | 0.861 |  | 0.797 | 0.802 | 0.594 | 0.872 |
| **CatBoost** | 0.764 | 0.760 | 0.528 | 0.846 |  | 0.784 | 0.783 | 0.569 | 0.860 |
| **BLOS-Khib** | **0.818** | **0.825** | **0.640** | **0.902** |  | **0.823** | **0.837** | **0.653** | **0.913** |

| **Table S19.** Performance comparison of machine learning classifiers with BLOS-Khib for Khib site prediction on the wheat dataset. | | | | | | | | | |
| --- | --- | --- | --- | --- | --- | --- | --- | --- | --- |
| **Classifier** | **10-fold cross-validation** | | | |  | **Independent test set** | | | |
|  | **ACC** | **F1** | **MCC** | **AUC** |  | **ACC** | **F1** | **MCC** | **AUC** |
| **KNN** | 0.559 | 0.575 | 0.119 | 0.577 |  | 0.543 | 0.560 | 0.085 | 0.564 |
| **SVM** | 0.663 | 0.660 | 0.326 | 0.724 |  | 0.669 | 0.668 | 0.339 | 0.727 |
| **RF** | 0.721 | 0.716 | 0.441 | 0.792 |  | 0.725 | 0.720 | 0.451 | 0.786 |
| **XGBoost** | 0.748 | 0.745 | 0.497 | 0.830 |  | 0.749 | 0.748 | 0.499 | 0.828 |
| **LightGBM** | 0.750 | 0.749 | 0.501 | 0.833 |  | 0.744 | 0.743 | 0.488 | 0.822 |
| **CatBoost** | 0.755 | 0.744 | 0.512 | 0.837 |  | 0.749 | 0.740 | 0.502 | 0.831 |
| **BLOS-Khib** | **0.810** | **0.817** | **0.626** | **0.890** |  | **0.790** | **0.777** | **0.586** | **0.892** |

| **Table S20.** Performance comparison of machine learning classifiers with BLOS-Khib for Khib site prediction on the *T.* *gondii* dataset. | | | | | | | | | |
| --- | --- | --- | --- | --- | --- | --- | --- | --- | --- |
| **Classifier** | **10-fold cross-validation** | | | |  | **Independent test set** | | | |
|  | **ACC** | **F1** | **MCC** | **AUC** |  | **ACC** | **F1** | **MCC** | **AUC** |
| **KNN** | 0.592 | 0.610 | 0.185 | 0.631 |  | 0.614 | 0.629 | 0.231 | 0.655 |
| **SVM** | 0.694 | 0.694 | 0.389 | 0.765 |  | 0.717 | 0.711 | 0.434 | 0.787 |
| **RF** | 0.729 | 0.718 | 0.459 | 0.805 |  | 0.741 | 0.733 | 0.482 | 0.815 |
| **XGBoost** | 0.745 | 0.735 | 0.491 | 0.822 |  | 0.758 | 0.745 | 0.516 | 0.828 |
| **LightGBM** | 0.744 | 0.738 | 0.440 | 0.823 |  | 0.759 | 0.750 | 0.517 | 0.829 |
| **CatBoost** | 0.750 | 0.730 | 0.506 | 0.830 |  | 0.761` | 0.741 | 0.525 | 0.830 |
| **BLOS-Khib** | **0.815** | **0.822** | **0.634** | **0.896** |  | **0.804** | **0.800** | **0.609** | **0.893** |

| **Table S21.** Performance comparison of machine learning classifiers with BLOS-Khib for Khib site prediction on the rice dataset. | | | | | | | | | |
| --- | --- | --- | --- | --- | --- | --- | --- | --- | --- |
| **Classifier** | **10-fold cross-validation** | | | |  | **Independent test set** | | | |
|  | **ACC** | **F1** | **MCC** | **AUC** |  | **ACC** | **F1** | **MCC** | **AUC** |
| **KNN** | 0.548 | 0.568 | 0.097 | 0.574 |  | 0.591 | 0.621 | 0.179 | 0.608 |
| **SVM** | 0.669 | 0.665 | 0.338 | 0.732 |  | 0.668 | 0.672 | 0.337 | 0.736 |
| **RF** | 0.706 | 0.701 | 0.412 | 0.771 |  | 0.706 | 0.711 | 0.413 | 0.773 |
| **XGBoost** | 0.733 | 0.732 | 0.466 | 0.803 |  | 0.715 | 0.722 | 0.430 | 0.794 |
| **LightGBM** | 0.736 | 0.736 | 0.473 | 0.807 |  | 0.730 | 0.738 | 0.459 | 0.805 |
| **CatBoost** | 0.731 | 0.722 | 0.463 | 0.798 |  | 0.730 | 0.731 | 0.461 | 0.795 |
| **BLOS-Khib** | **0.785** | **0.794** | **0.577** | **0.869** |  | **0.807** | **0.822** | **0.614** | **0.887** |

| **Table S22.** Performance comparison of machine learning classifiers with BLOS-Khib for Khib site prediction on the Candida dataset. | | | | | | | | | |
| --- | --- | --- | --- | --- | --- | --- | --- | --- | --- |
| **Classifier** | **10-fold cross-validation** | | | |  | **Independent test set** | | | |
|  | **ACC** | **F1** | **MCC** | **AUC** |  | **ACC** | **F1** | **MCC** | **AUC** |
| **KNN** | 0.569 | 0.570 | 0.137 | 0.598 |  | 0.559 | 0.562 | 0.118 | 0.592 |
| **SVM** | 0.693 | 0.693 | 0.385 | 0.760 |  | 0.687 | 0.687 | 0.375 | 0.773 |
| **RF** | 0.734 | 0.733 | 0.468 | 0.810 |  | 0.727 | 0.722 | 0.455 | 0.816 |
| **XGBoost** | 0.752 | 0.755 | 0.504 | 0.835 |  | 0.761 | 0.760 | 0.522 | 0.843 |
| **LightGBM** | 0.755 | 0.756 | 0.510 | 0.837 |  | 0.771 | 0.768 | 0.544 | 0.858 |
| **CatBoost** | 0.747 | 0.739 | 0.494 | 0.827 |  | 0.754 | 0.742 | 0.513 | 0.837 |
| **BLOS-Khib** | **0.804** | **0.809** | **0.611** | **0.886** |  | **0.801** | **0.803** | **0.602** | **0.885** |

| **Table S23.** Performance comparison of machine learning classifiers with BLOS-Khib for Khib site prediction on the *B.* *cinerea* dataset. | | | | | | | | | |
| --- | --- | --- | --- | --- | --- | --- | --- | --- | --- |
| **Classifier** | **10-fold cross-validation** | | | |  | **Independent test set** | | | |
|  | **ACC** | **F1** | **MCC** | **AUC** |  | **ACC** | **F1** | **MCC** | **AUC** |
| **KNN** | 0.573 | 0.598 | 0.148 | 0.598 |  | 0.573 | 0.619 | 0.134 | 0.600 |
| **SVM** | 0.665 | 0.655 | 0.329 | 0.729 |  | 0.667 | 0.685 | 0.333 | 0.749 |
| **RF** | 0.709 | 0.695 | 0.418 | 0.785 |  | 0.712 | 0.717 | 0.434 | 0.801 |
| **XGBoost** | 0.737 | 0.734 | 0.474 | 0.812 |  | 0.767 | 0.782 | 0.533 | 0.840 |
| **LightGBM** | 0.740 | 0.734 | 0.479 | 0.812 |  | 0.741 | 0.756 | 0.483 | 0.824 |
| **CatBoost** | 0.745 | 0.730 | 0.492 | 0.820 |  | 0.727 | 0.732 | 0.462 | 0.836 |
| **BLOS-Khib** | **0.800** | **0.806** | **0.604** | **0.882** |  | **0.819** | **0.833** | **0.635** | **0.903** |

| 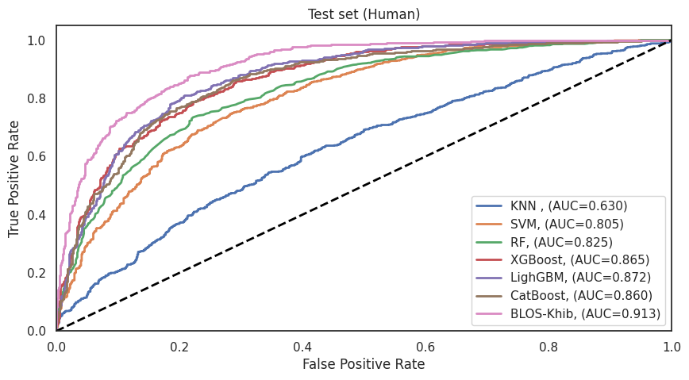 | 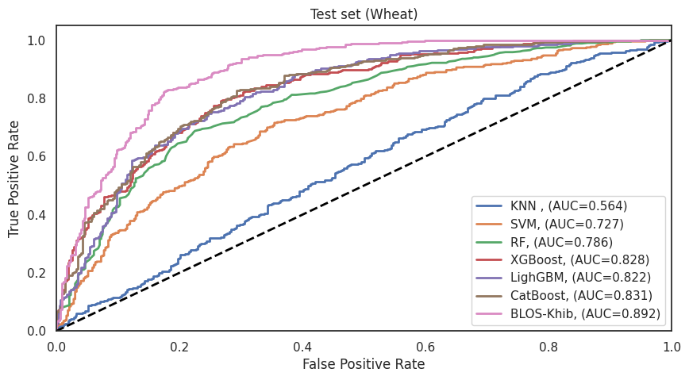 |
| --- | --- |
| (a) | (b) |
| 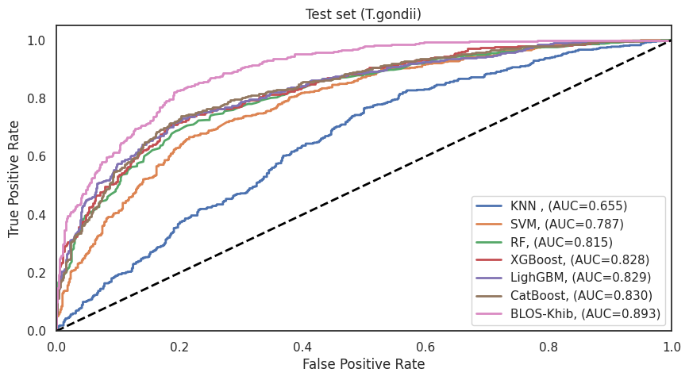 | 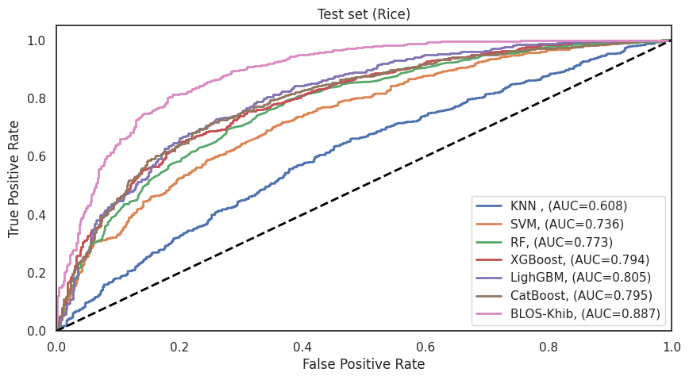 |
| (c) | (d) |
| 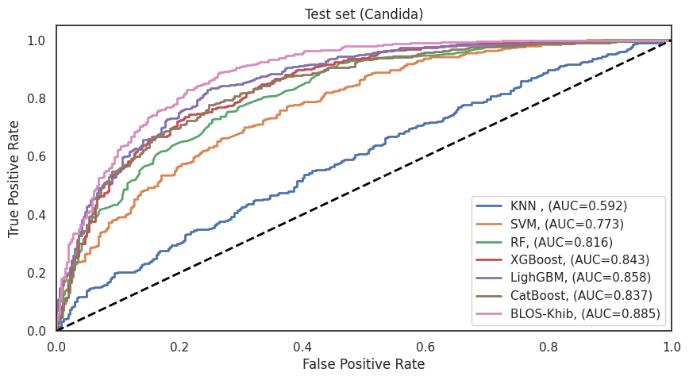 | 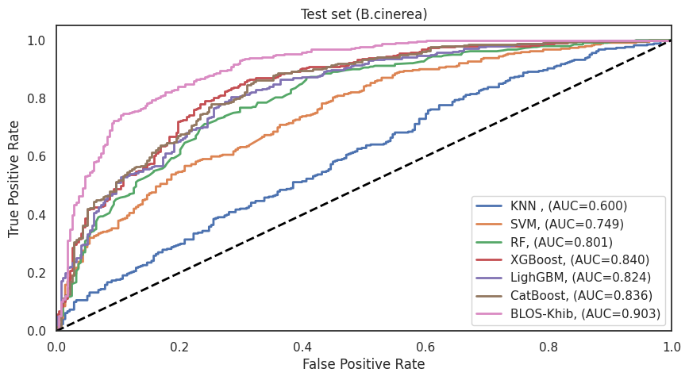 |
| (e) | (f) |
| **Fig. S4** ROC curves comparing machine learning classifiers with BLOS-Khib on the independent test sets from six datasets: (a) human, (b) wheat, (c) *T. gondii*, (d) rice, (e) Candida, and (f) *B. cinerea*. | |
